# Systematic assessment of tissue dissociation and storage biases in single-cell and single-nucleus RNA-seq workflows

**DOI:** 10.1186/s13059-020-02048-6

**Published:** 2020-06-02

**Authors:** Elena Denisenko, Belinda B. Guo, Matthew Jones, Rui Hou, Leanne de Kock, Timo Lassmann, Daniel Poppe, Olivier Clément, Rebecca K. Simmons, Ryan Lister, Alistair R. R. Forrest

**Affiliations:** 1 Harry Perkins Institute of Medical Research, QEII Medical Centre and Centre for Medical Research, the University of Western Australia, PO Box 7214, 6 Verdun Street, Nedlands, Perth, Western Australia 6009 Australia; 2 Telethon Kids Institute, Perth’s Children Hospital, the University of Western Australia, 15 Hospital Avenue, Nedlands, Perth, Western Australia 6009 Australia; 3 Australian Research Council Centre of Excellence in Plant Energy Biology, School of Molecular Sciences, the University of Western Australia, 35 Stirling Hwy, Crawley, Perth, Western Australia 6009 Australia

**Keywords:** Single-cell transcriptomics, RNA-seq, scRNA-seq, snRNA-seq, 10x Genomics

## Abstract

**Background:**

Single-cell RNA sequencing has been widely adopted to estimate the cellular composition of heterogeneous tissues and obtain transcriptional profiles of individual cells. Multiple approaches for optimal sample dissociation and storage of single cells have been proposed as have single-nuclei profiling methods. What has been lacking is a systematic comparison of their relative biases and benefits.

**Results:**

Here, we compare gene expression and cellular composition of single-cell suspensions prepared from adult mouse kidney using two tissue dissociation protocols. For each sample, we also compare fresh cells to cryopreserved and methanol-fixed cells. Lastly, we compare this single-cell data to that generated using three single-nucleus RNA sequencing workflows. Our data confirms prior reports that digestion on ice avoids the stress response observed with 37 °C dissociation. It also reveals cell types more abundant either in the cold or warm dissociations that may represent populations that require gentler or harsher conditions to be released intact. For cell storage, cryopreservation of dissociated cells results in a major loss of epithelial cell types; in contrast, methanol fixation maintains the cellular composition but suffers from ambient RNA leakage. Finally, cell type composition differences are observed between single-cell and single-nucleus RNA sequencing libraries. In particular, we note an underrepresentation of T, B, and NK lymphocytes in the single-nucleus libraries.

**Conclusions:**

Systematic comparison of recovered cell types and their transcriptional profiles across the workflows has highlighted protocol-specific biases and thus enables researchers starting single-cell experiments to make an informed choice.

## Background

Single-cell RNA sequencing (scRNA-seq) is an increasingly powerful technology that enables analysis of gene expression in individual cells. ScRNA-seq has been recently used to study organism development [1–3], normal tissues [4–6], cancer [7–10], and other diseases [11, 12]. These studies have shed light on tissue heterogeneity and provided previously inaccessible insights into tissue functioning.

Advances in high-throughput droplet-based microfluidics technologies have facilitated analysis of thousands of cells in parallel [13–15], and Chromium from 10x Genomics has become a widely used commercial platform [15]. Multiple tissue preparation protocols are compatible with Chromium, but the protocol of choice should ideally maintain RNA integrity and cell composition of the original tissue.

Solid tissues need to be dissociated to release individual cells suitable for 10x Genomics Chromium scRNA-seq. However, optimal dissociation needs to achieve a balance between releasing cell types that are difficult to dissociate while avoiding damage to those that are fragile. Tissue dissociation is most commonly conducted using enzymes which require incubation at 37 °C for variable times based on tissue type. At this temperature, the cell transcriptional machinery is active; hence, gene expression can be altered in response to the dissociation and other environmental stresses [16, 17]. A recent alternative approach minimizing this artifact uses cold-active protease to conduct tissue dissociation on ice [18]. Alternatively, single-nucleus RNA sequencing protocols (snRNA-seq) use much harsher conditions to release nuclei from tissue and can be applied to snap frozen samples, thus avoiding many of the dissociation-related artifacts [19, 20]. Single-nuclei methods should also permit profiling of nuclei from large cells (> 40 μm) that do not fit through the microfluidics.

Additional restrictions and challenges are faced by complex experimental designs where specimens cannot be processed immediately. In this case, samples need to be preserved either as an intact tissue or in a dissociated form as a single-cell suspension. Each of the approaches mentioned above introduces specific biases and artifacts that can manifest themselves in altered transcriptional profiles or altered representation of cell types. These biases need to be considered when designing and analyzing data from a single-cell experiment; however, they are still incompletely understood.

Some of the artifacts have been investigated in recent studies comparing single-cell profiles of methanol-fixed and live cells [21, 22], cryopreserved and live cells [22, 23], single-cell and single-nucleus protocols [24–26], or tissue dissociation using cold-active protease and traditional digestion at 37 °C [18]. However, these assessments were performed in different tissues under different conditions and lack extensive comparison to bulk data.

**Fig. 1:**
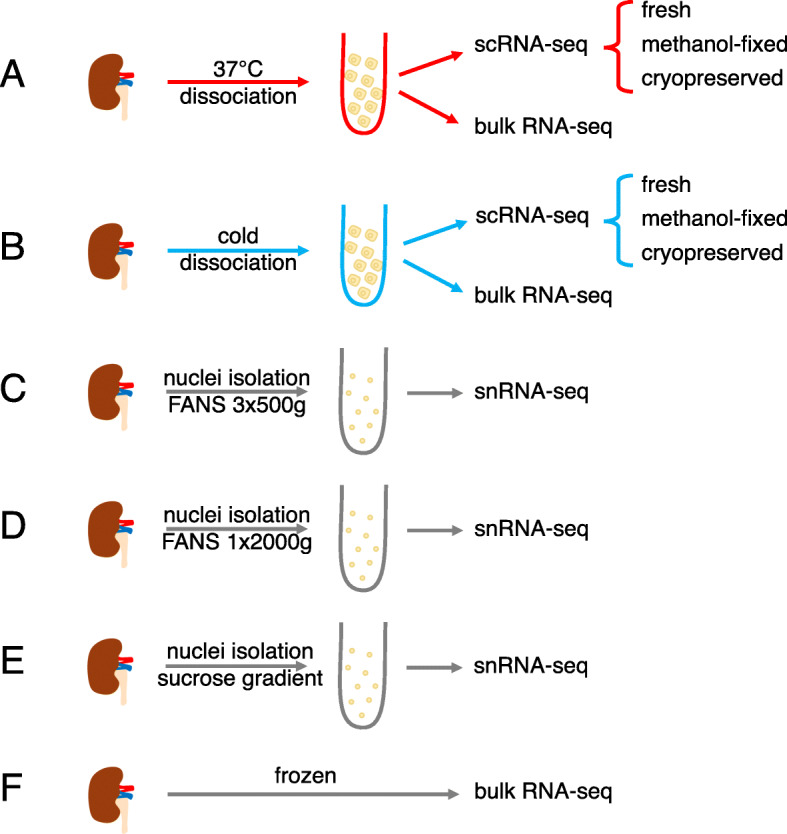
Overview of experiments performed in this study. All experiments were carried out in biological triplicate using three kidneys from three different mice. **a** 37 °C dissociation used the Multi-tissue dissociation kit 2 from Miltenyi Biotec. **b** Cold dissociation was carried out on ice using *B. Licheniformis* protease. In **a** and **b**, methanol-fixed samples used 80% MeOH at − 20 °C and then were stored at − 80 °C. Cryopreservation was carried out using 50% FBS, 40% RPMI-1640, 10% and DMSO with gradient cooling to − 80 °C then stored in liquid nitrogen. **c–e** Whole kidneys were flash frozen using an isopentane bath − 30 °C and then stored at − 80 °C. Three different nuclei preparation methods were tested using either fluorescently activated nuclei sorting (FANS) or a sucrose gradient to enrich for singlet nuclei. **f** Bulk RNA-seq was carried out using the NEBNext Ultra II RNA Library Kit for Illumina with rRNA depletion or NEBNext Poly(A) mRNA isolation module. See “Methods” for more details

Here, we performed a comprehensive study in healthy adult mouse kidneys using 10x Genomics Chromium workflows for scRNA-seq and snRNA-seq, along with bulk RNA-seq of undissociated and dissociated tissue. We compare and contrast two tissue dissociation protocols (digestion at 37 °C, further referred to as warm dissociation, or with cold-active protease, further referred to as cold dissociation), two single-cell suspension preservation methods (methanol fixation and cryopreservation) and three single-nuclei isolation protocols (Fig. 1). A total of 77,656 single-cell, 98,303 single-nucleus, and 15 bulk RNA-seq profiles were generated and made publicly available (GSE141115). Our dissection of artifacts associated with each of the approaches will serve as a valuable resource to aid interpretation of single-cell and single-nucleus gene expression data and help to guide the choice of experimental workflows.

## Results

### Comparison of tissue dissociation protocols

In the first series of experiments, we set out to compare two tissue dissociation protocols using kidneys from adult male C57BL/6J mice. Kidneys were dissociated at 37 °C using a commercial Miltenyi Multi Tissue Dissociation Kit 2 or on ice using a cold-active protease from *Bacillus licheniformis* (“Methods”, Fig. 1a, b). Aliquots of single-cell suspensions were profiled using 10x Genomics Chromium scRNA-seq and a bulk RNA-seq protocol (“Methods”, Fig. 1a, b). All experiments were performed in triplicate, and data were processed as described in “Methods”.

#### Warm tissue dissociation induces stress response

**Fig. 2:**
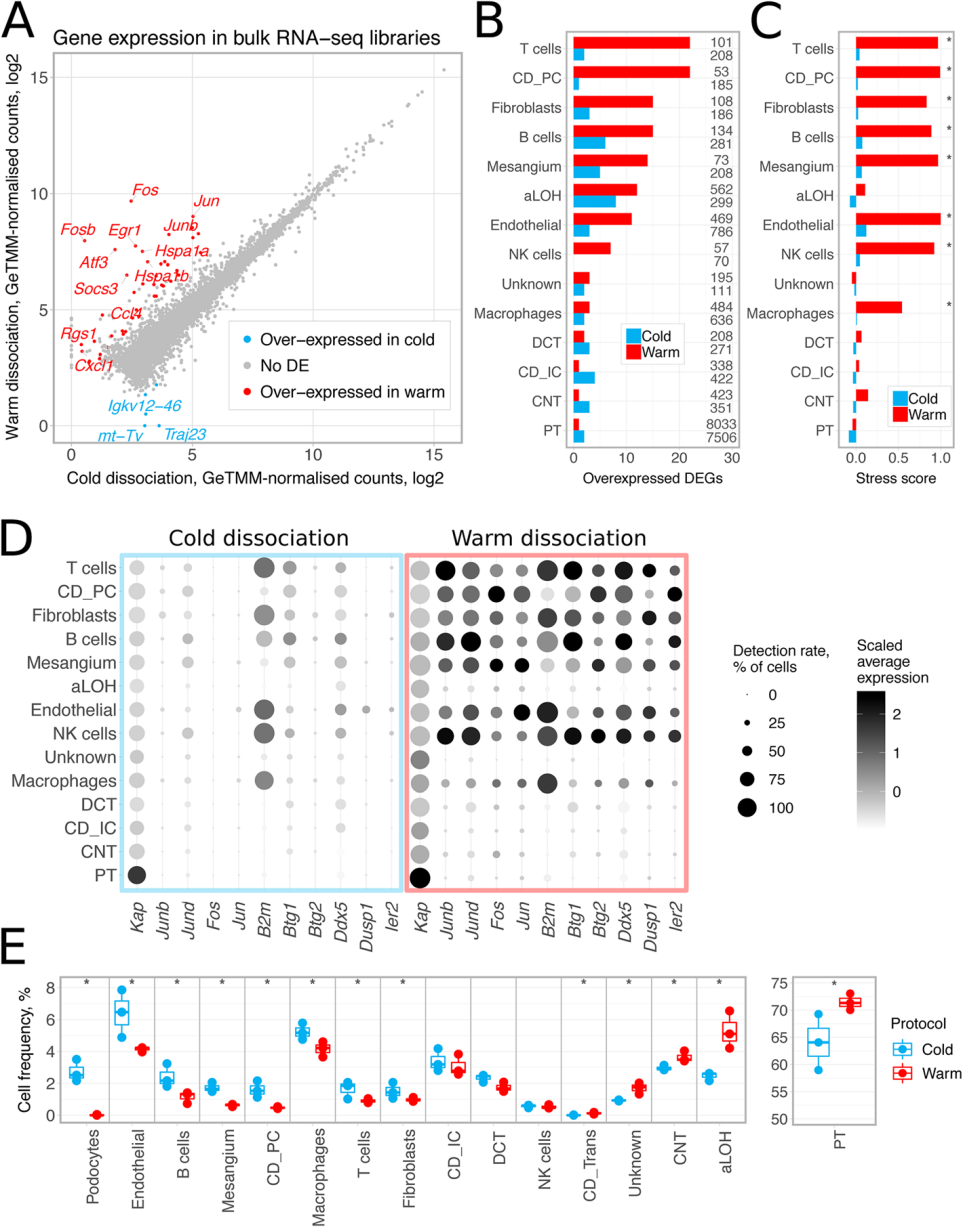
Comparison of cold and warm tissue dissociation protocols. **a** Bulk RNA-seq profiles of dissociated kidneys. GeTMM-normalized counts [29] were averaged across three biological replicates and log2-transformed after adding a pseudo count of 1. DEGs with FDR < 0.05 and logFC threshold of 2 (edgeR exact test [27]) are shown as red and blue dots; protein-coding genes with logFC > 4 are labelled. **b** Number of differentially expressed genes (DEGs) between cold- and warm-dissociated scRNA-seq libraries. Calculated for each cell type separately using Wilcoxon test in Seurat [30] with thresholds of logFC = 0.5, minimum detection rate 0.5, FDR < 0.05. Numbers on the right side of the plot indicate cell population size. **c** Stress score – an expression score for a set of 17 stress-response-related genes (*Fosb*, *Fos*, *Jun*, *Junb*, *Jund*, *Atf3*, *Egr1*, *Hspa1a*, *Hspa1b*, *Hsp90ab1*, *Hspa8*, *Hspb1*, *Ier3*, *Ier2*, *Btg1*, *Btg2*, *Dusp1*). Calculated as average gene expression level of these genes subtracted by averaged expression of randomly selected control genes and then averaged for cell types. Significance was calculated in a Monte-Carlo procedure with 1000 randomly selected gene sets of the same size, asterisks denote *p* value < 0.01. **d** Expression and detection rates of differentially expressed genes commonly induced in warm-dissociated samples (differentially expressed in at least four cell types). **e** Cell type composition of freshly profiled scRNA-seq libraries. Three biological replicates are shown per condition. Asterisks denote two-sided chi-square test *p* value < 0.001. In **b–d**, podocytes and transitional cells were excluded due to low cell numbers. aLOH: ascending loop of Henle; CD_IC: intercalated cells of collecting duct; CD_PC: principal cells of collecting duct; CNT: connecting tubule; DCT: distal convoluted tubule; PT: proximal tubule

Bulk RNA-seq profiling of single-cell suspensions revealed induction of stress response genes in warm-dissociated samples. Differential expression analysis identified 71 genes with higher expression in warm-dissociated kidneys and 5 genes with higher expression in cold-dissociated kidneys (logFC > 2, FDR < 0.05, edgeR exact test [27], Additional file 1). Gene ontology analysis with ToppGene [28] reported “regulation of cell death” as the top significantly enriched biological process for the genes more highly expressed in warm-dissociated kidneys (an overlap of 22 genes, FDR = 1.7E−7, see Additional file 1). Genes with the highest logFC values (> 4) included immediate-early genes *Fosb*, *Fos*, *Jun*, *Junb*, *Atf3*, and *Egr1* and heat shock proteins *Hspa1a* and *Hspa1b* (Fig. 2a). These findings from bulk RNA-seq confirm the original observations of Adam et al. [18] that warm tissue dissociation induces substantial stress-response-related changes.

#### Single-cell sequencing reveals heterogeneous stress response across cell populations

We next characterized differences between the two tissue dissociation protocols by scRNA-seq profiling of fresh cell suspensions (Fig. 1a, b). This dataset comprised 23,108 cells, including 11,851 cells from cold- and 11,257 cells from warm-dissociated kidneys (“Methods”). Cells were classified into 15 cell types using scMatch [31] by comparing their expression to reference expression profiles from three previous mouse kidney studies [26, 32, 33], followed by gene signature-based refinement (“Methods”, Additional file 2: Figure S1–3, Additional file 3).

Differential expression analysis identified 64 genes more highly expressed in warm-dissociated libraries in at least one cell type (Fig. 2b, Additional file 4) and gene ontology analysis again reported “regulation of cell death” as one of the top significantly enriched terms (an overlap of 23 genes, FDR = 3.9E−7, Additional file 4). The genes most commonly overexpressed across cell populations are shown in Fig. 2d and include immediate-early response genes such as *Junb* and *Jund* (differentially expressed in seven cell types) and *Jun* and *Fos* (differentially expressed in five cell types).

Notably, the numbers of differentially expressed genes varied among cell types (Fig. 2b), suggesting that cell types respond differently to warm tissue dissociation. To quantify these differences, we selected a set of 17 known stress-response-related genes that were induced in the warm-dissociated samples (*Fosb*, *Fos*, *Jun*, *Junb*, *Jund*, *Atf3*, *Egr1*, *Hspa1a*, *Hspa1b*, *Hsp90ab1*, *Hspa8*, *Hspb1*, *Ier3*, *Ier2*, *Btg1*, *Btg2*, *Dusp1*) and used them to calculate a stress score (see “Methods”). Figure 2c shows that significantly high stress scores were detected only in warm-dissociated samples in eight out of 14 cell types. Taken together, these results highlight that certain cell types, such as immune and endothelial cells, are particularly sensitive to warm tissue dissociation.

In contrast to the 64 genes with higher expression in the warm dissociation, only 20 genes had higher expression in the cold-dissociated cell populations, and only five of them (*Hbb-bs*, *Hba-a1*, *Hba-a2*, *mt-Co1*, *Malat1*) were identified in at least two cell types (Fig. 2b, Additional file 5). We note the levels of hemoglobin transcripts suggest contamination from erythrocytes is higher in the samples dissociated on ice.

#### Cell composition differs between two tissue dissociation protocols

In addition to expression changes, our analyses identified eight cell populations that were less abundant in warm-dissociated samples in comparison to cold-dissociated ones, including podocytes, mesangial cells, and endothelial cells (Fig. 2e left, chi-square test *p* value < 0.001). These depleted populations also showed significantly high expression of the stress-response-related gene set as described above (Fig. 2c). Notably, only three podocytes were detected in warm-dissociated samples (0.03% of the total cell count), compared to 330 (2.78%) in the cold-dissociated samples. These findings suggest that these populations are sensitive to warm dissociation and consequently underrepresented.

Conversely, we identified cells such as those of the ascending loop of Henle (aLOH) and proximal tubule (PT), that were more abundant in warm-dissociated samples (aLOH 4.99% vs. 2.52% in cold, PT 71.36% vs. 63.34% in cold), potentially indicating their less efficient dissociation by cold-active protease (Fig. 2e right).

Finally, to determine whether microfluidic partitioning could affect cell composition, we compared bulk RNA-seq and scRNA-seq data generated on the same dissociated kidney samples (Additional file 6: Supplementary Note 1). Our results suggest that proportions of aLOH cells in cell suspensions were higher before they were loaded on the Chromium Controller and that they are somehow underrepresented. As to possible mechanisms for this, it may be that they are more resistant to lysis in the device or that there is differential sampling of these cells due to cell size or shape as they enter the microfluidic device.

### Comparison of cell preservation protocols

We next evaluated whether cryopreservation and methanol fixation maintain cell composition and transcriptional profiles of kidneys. Aliquots of single-cell suspensions of cold- and warm-dissociated kidneys were cryopreserved (50% FBS, 40% RPMI-1640, 10% DMSO) and stored for 6 weeks or methanol-fixed and stored for 3 months. These stored samples were then profiled with 10x Genomics Chromium scRNA-seq (Fig. 1a, b, “Methods”). The resulting datasets consisted of 11,627 and 5545 methanol-fixed cells and 3519 and 3483 cryopreserved cells derived from cold- and warm-dissociated kidneys, respectively. Despite loading similar numbers of cells, the number of high-quality cells obtained from the cryopreserved samples after quality control and filtering (“Methods”) was substantially lower (~ 30%) than that of the fresh and methanol-fixed samples.

#### Cryopreservation depletes epithelial cell types

**Fig. 3:**
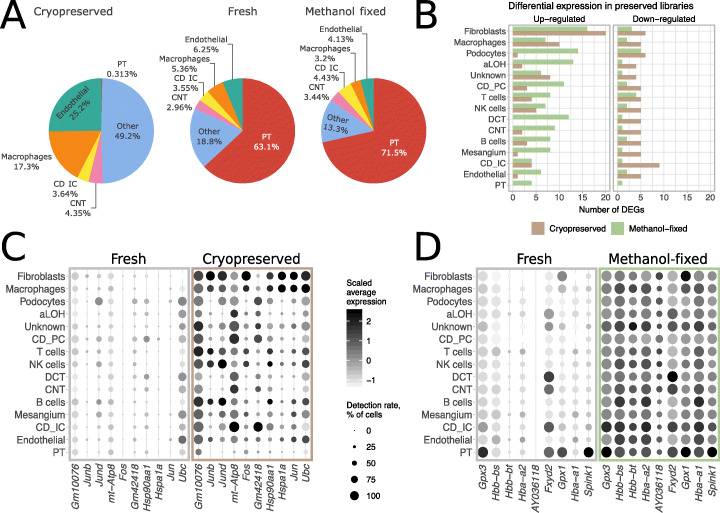
Cell preservation protocol performance in cold-dissociated samples. **a** Cell type composition of freshly profiled and preserved cold-dissociated samples. **b** Number of differentially expressed genes (DEGs) detected between preserved and freshly profiled aliquots. Seurat Wilcoxon test [30] with logFC = 1, min detection rate 0.5, FDR < 0.05 as thresholds. **c** Expression and detection rates of DEGs with higher expression in cryopreserved samples in at least two cell types. **d** Expression and detection rates of DEGs with higher expression in methanol-fixed samples in at least nine cell types. aLOH: ascending loop of Henle; CD_IC: intercalated cells of collecting duct; CD_PC: principal cells of collecting duct; CNT: connecting tubule; DCT: distal convoluted tubule; PT: proximal tubule

The most prominent difference in recovery rates pertained to cells of the proximal tubule (PT), the most populous cell type in kidney [34]. In freshly profiled suspensions, PT composed 63.12% and 70.86% of all cells in cold- and warm-dissociated samples, respectively. In contrast, PT were scarcely detected in cryopreserved samples, at 0.31% and 0.57%, respectively (see Fig. 3a for cold-dissociated samples, Additional file 2: Figure S4 for warm-dissociated samples, Additional file 2: Figure S5 for biological replicates). We next compared recovery rates of other cell populations in freshly profiled and cryopreserved samples relative to all non-PT cells. This comparison revealed significant underrepresentation (chi-square test *p* value < 0.001) of five kidney cell types in cryopreserved samples prepared with the cold dissociation protocol, three of which were also underrepresented in the cryopreserved warm dissociation samples (Additional file 2: Figure S6). Together with the loss of PT cells, this indicates that the cryopreservation and subsequent thawing protocol failed to efficiently recover kidney epithelial cell populations.

Previous studies have reported cryopreserved cells generate comparable data to that of fresh cells [22, 23]. Hence, we repeated the experiment comparing cryopreserved and freshly profiled cold-dissociated single-cell suspension aliquots using different mice (Balb/c female), 10x chemistry (v3 as opposed to v2), storage length (2 weeks as opposed to 6 weeks), and centrifugation speed for thawing and resuspension (1200*g* as opposed to 400*g*). Again, there was a significant depletion of PT cells, with them making up 55.55% of the freshly profiled cells but only 7.65% of the cryopreserved cells (Additional file 2: Figure S7). Notably, only ~ 33% and ~ 32% of cells were recovered after cryostorage in the first and the repeated experiment, respectively; the average viability estimated by the Countess was 86% and 75%, respectively. From this, we conclude that, at least in the case of mouse kidneys, cryopreservation of dissociated cells using 50% FBS, 40% RPMI, and 10% DMSO can induce substantial deleterious changes in cell composition.

In contrast to a previous report assessing storage of cell lines and immune cells [22], in the case of dissociated mouse kidneys, methanol fixation better preserved cell type composition than cryopreservation (Fig. 3a, Additional file 2: Figure S4–5). Nevertheless, certain cell types were moderately underrepresented in the methanol-fixed samples in comparison to freshly profiled samples, with macrophages showing the largest reduction from 5.36 to 3.2% in cold-dissociated samples and from 4.28 to 2.54% in warm-dissociated samples.

#### Cryopreservation induces stress response

To gain further insights into preservation-related artifacts, we compared gene expression between preserved and freshly profiled samples in each cell type separately. In cold-dissociated samples, 31 and 27 genes were overexpressed in at least one cell type in cryopreserved and methanol-fixed cells, respectively, when compared to freshly profiled suspensions (Fig. 3b, see Additional file 2: Figure S4 for warm-dissociated samples).

In cryopreserved samples, stress-response-related genes were induced, including multiple immediate-early response genes and heat shock proteins (Fig. 3c, Additional file 2: Figure S4, Additional files 7, 8). In contrast, genes overexpressed in methanol-fixed cells were those highly expressed in tubular cells and hemoglobin genes (Fig. 3d, Additional files 9, 10). The same set of transcripts contaminated most cell types suggesting methanol fixation damages cells and leads to ambient RNA contamination of droplets (see Additional file 6: Supplementary Note 2 for more detailed investigation of the ambient RNA profile).

### Comparison of single-cell and single-nucleus sequencing protocols

Having identified cold-active protease as a less damaging tissue dissociation approach for scRNA-seq, we next compared it to snRNA-seq. We performed a series of experiments using kidneys from Balb/c male mice with v2 10x chemistry or female mice with v3 chemistry and prepared cells using cold tissue dissociation for scRNA-seq and nuclei using three variant protocols for snRNA-seq (“Methods”, Fig. 1b–e). Two nuclei isolation protocols made use of fluorescence-activated nuclei sorting (FANS). The first protocol washed the nuclei three times and used a centrifugation speed of 500*g* (further referred to as SN_FANS_3x500g, Fig. 1c). In the second protocol, nuclei were washed once and a centrifugation speed of 2000*g* was used (SN_FANS_1x2000g, Fig. 1d). In the third protocol, nuclei were initially washed using a 500*g* spin and then cleaned using a sucrose cushion avoiding the requirement to sort isolated nuclei (SN_sucrose, Fig. 1e). The three nuclei isolation protocols yielded comparable results, with the most notable difference being a higher contamination with mitochondrial genes in SN_FANS_1x2000g (see Additional file 6: Supplementary Note 3 and Additional files 11, 12, 13). Single-nuclei sequencing detected more genes per nuclei than single-cell sequencing per cell, with the median numbers of 1819 and 981 genes, respectively (genes detected in at least 10 cells/nuclei were retained in each sample). In addition, we performed bulk RNA-seq of intact flash-frozen whole kidneys and of cold-dissociated cell suspensions (Fig. 1b, f).

**Fig. 4:**
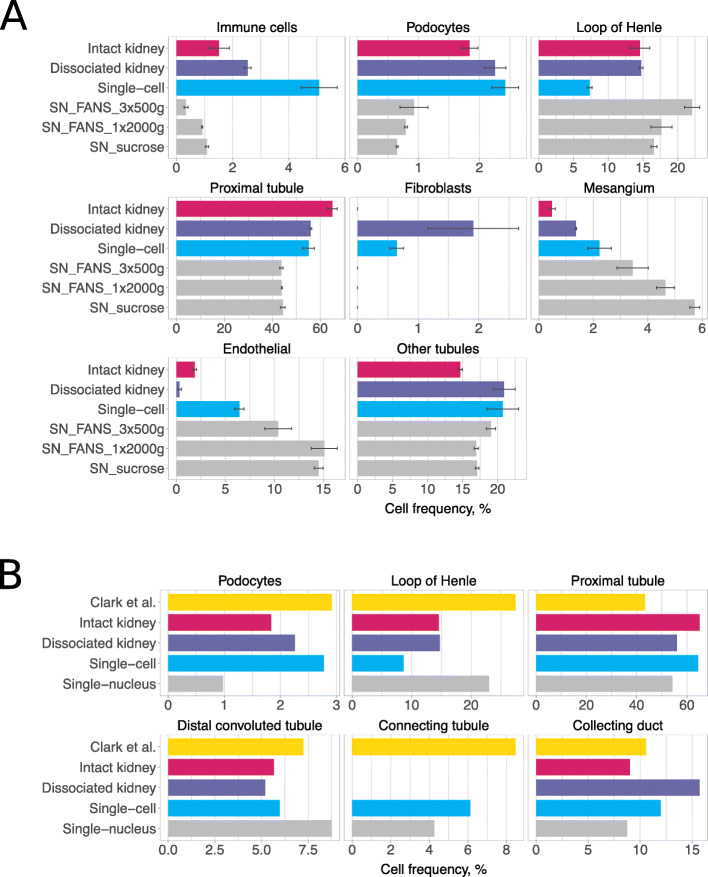
Comparison of single-cell and single-nucleus libraries. **a** Cell type composition for kidneys from Balb/c female mice. Average percentages for scRNA-seq libraries are shown in blue and for snRNA-seq libraries in gray. BSEQ-sc estimates are shown for bulk RNA-seq of intact and dissociated kidneys. Error bars are standard error of mean. **b** Abundance of renal epithelial cell types in Clark et al. study [34] in comparison to our data from Balb/c female mice

Detection rates of non-epithelial kidney cell types were markedly different between scRNA-seq and snRNA-seq libraries (Fig. 4a, Additional file 2: Figure S8, Additional file 14). Immune cells were detected at lower rates in snRNA-seq (average of 0.73%) than in scRNA-seq (average of 6.03%) across all experiments performed (Fig. 4a, Additional file 2: Figure S8). Using the bulk RNA-seq from intact kidneys (Fig. 1f) and BSEQ-sc [35] to predict the proportions of each cell type present, we estimated that approximately 1.51% should correspond to immune cells in Balb/c female mice and 4.84% in Balb/c male mice. This suggests an underrepresentation of immune cells in the snRNA-seq data. Furthermore, macrophages were the only type of immune cells recovered in snRNA-seq libraries, whereas in scRNA-seq libraries we also detected T cells (1.38% on average), B cells (0.77%), and NK cells (0.65%). Similarly, podocytes composed only 0.7% in snRNA-seq libraries as opposed to 3.28% in scRNA-seq (Fig. 4a, Additional file 2: Figure S8). Cell types more abundant in snRNA-seq libraries included loop of Henle and mesangial cells (Fig. 4a, Additional file 2: Figure S8).

We next compared the observed cell composition to estimates of epithelial cell type contribution based on quantitative renal anatomy, as reported by Clark et al. recently [34]. Figure 4b and Additional file 2: Figure S8 show that for some cell types, such as podocytes, scRNA-seq yields proportions most similar to the quantitative renal anatomy estimates, whereas for other cell types, such as loop of Henle cells, snRNA-seq better captures cell composition. Bulk RNA-seq-based proportions estimated from intact kidneys largely contradicted the anatomical estimates, which might reflect inaccurate deconvolution of the sample. Finally, comparison of bulk RNA-seq profiles of intact kidneys and cold-dissociated cell suspensions from female Balb/c mice suggested cell types that may be unequally represented in whole vs dissociated kidneys (see Additional file 6: Supplementary Note 4 and Additional file 15).

Differential expression analysis comparing individual cell types profiled by snRNA-seq and scRNA-seq suggested higher expression of long noncoding RNAs in snRNA-seq libraries and higher expression of genes related to mitochondrial and ribosomal functions in scRNA-seq, in agreement with previous reports [24, 26] (Additional file 16).

Finally, as mitotic cells lack a nuclear membrane and in principle should not be observed in the snRNA-seq data, we inferred cell cycle phases for cells and nuclei using Seurat (“Methods”) [30]. Notably, Seurat predicted a higher fraction of G1 phase cells and lower fraction of S phase cells in scRNA-seq libraries when compared to snRNA-seq libraries for virtually all cell types (Additional file 2: Figure S9). This suggests that there are indeed underlying biases in cell cycle phase distributions in snRNA-seq data in comparison to scRNA-seq data; however, to fully dissect this, a classifier that can discriminate mitotic cells from early G1 and late G2 is required.

## Discussion

Interrogating complex tissues at the level of individual cells is essential to understand organ development, homeostasis, and pathological changes. Despite the rapid advancement and widespread adoption of scRNA-seq and snRNA-seq technologies, the associated biases remain incompletely understood. To characterize some of the biases, we performed a systematic comparison of recovered cell types and their transcriptional profiles across two tissue dissociation protocols, two single-cell suspension preservation methods and three single-nuclei isolation protocols, each with three biological replicates per experiment.

Previous studies have reported on artifactual gene expression changes induced by proteolytic tissue digestion at 37 °C in sensitive cell populations [16, 18]. Our findings corroborate this bias and show induction of heat shock proteins and immediate-early response genes in warm-dissociated libraries when compared to cold-dissociated libraries. Cold-dissociated libraries can serve as a baseline in this case, since low temperature should minimize new transcription [18]. Our results further indicate that cell populations prone to these transcriptional changes are also depleted from the samples, with podocytes being the extreme example of a cell type practically lost in warm-dissociated libraries. Overexpression of stress-response-related genes was also detected by bulk RNA-seq analysis of dissociated tissues, confirming that this artifact stems from the dissociation protocol rather than from microfluidic separation, single-cell sequencing, or data processing. These findings have important implications and suggest that data from samples digested at 37 °C needs to be interpreted in light of this bias.

One possible drawback of cold tissue dissociation is lower efficiency of releasing hard-to-dissociate cell types. In our study, this may have affected cells of loop of Henle, which were detected at 2.52% in cold- and at 4.99% in warm-dissociated samples. However, both protocols dramatically underestimated abundance of this second most populous kidney cell type. While one possible explanation could be incomplete tissue dissociation in both cases, deconvolution of bulk RNA-seq profiles of single-cell suspensions indicated that cells might be lost during cell encapsulation on the microfluidic device.

Cold-dissociated samples showed higher contamination with hemoglobin transcripts than warm-dissociated ones. We note that red blood cell depletion methods were not applied in any of the experiments performed here and the observation likely stems from a higher rate of hemoglobin transcript degradation in warm tissue dissociation conditions.

Two recent studies have shown that cryopreservation generated comparable data to that of fresh cells for cell lines and immune cells, and also for complex tissues cryopreserved prior to single-cell separation [22, 23]. Here, however, we report that cryopreservation of single-cell suspensions of dissociated mouse kidneys resulted in depletion of epithelial cell types. This artifact was reproducible across two mouse strains, both sexes, and two 10x chemistry versions. However, we observed a higher fraction of recovered PT cells (7.65% vs. 0.57%) in the repeated experiment, which might be explained by either sex or strain differences, or higher sensitivity of 10x v3 chemistry. Together with the depletion of PT cells, we observed reduced contamination of other cells with highly expressed PT transcripts, which indicates that PT cells might be lost in the thawing and resuspension. A possible explanation for the differences from previous reports is the proportion of serum used in the freezing media. 10x Genomics recommends 40% FBS (10x Genomics, CG00039, Rev. D), whereas the other studies used either 90% FBS (peripheral blood, minced tissues, cell lines, and immune cells) or 10% FBS (cell lines) [22, 23]. Notably, despite loading similar numbers of fresh, methanol-fixed and cryopreserved cells, the number of the usable cells observed in the cryopreserved samples was only ~ 30% of the others. This raises the possibilities that the missing PT cells may be present but are failing to make it into the microfluidics, failing to lyse, or are so badly damaged that there is insufficient RNA remaining to generate a usable library. In contrast to cryopreservation, cell composition of methanol-fixed suspensions resembled that of freshly profiled libraries. Similarly to previous studies, we observed ambient RNA contamination with highly abundant transcripts suggesting cell damage by methanol fixation [22].

Studies comparing scRNA-seq and snRNA-seq reported that, although the two technologies profile different RNA fractions, both detect sufficient genes and allow adequate representation of cell populations [24–26]. In this work, one of the most notable differences between single-cell and single-nuclei experiments was the low detection rate of immune cells, in particular the failure to detect T, B, or NK cells in any of the snRNA-seq libraries. The depletion of lymphocytes was also observed in the Wu et al. [26] dataset (commented upon by O’Sullivan et al. [36]). Notably Slyper et al. [37] also observe much lower fractions of T, B, and NK cells in matched snRNA-seq and scRNA-seq datasets from adjacent pieces of a metastatic breast cancer and a neuroblastoma. As Wu et al. have suggested, although these differences might indicate underestimation of immune cells by snRNA-seq, another plausible explanation is that immune cell content is inflated in single-cell experiments as other cell types may be underrepresented due to incomplete dissociation.

It is important to remember that cell type composition of single-cell or single-nucleus data differs from that of the original tissue and complementary approaches may be considered to improve the accuracy of the estimates. We note that even ISH/IHC/spatial transcriptomic methods, unless carried out on sufficient serial sections to completely survey the tissue of interest, will only give cellular proportions for the sections examined and will not give a whole (3D) organ estimate. Similarly, flow cytometry would help as an orthogonal approach to quantify cellular proportions within single-cell suspensions but still requires the organ to be dissociated, which as we have shown here has biases. Although in vitro cell mixtures or cell spiking experiments could be used to introduce different cell types at known ratios, we have not employed them in these experiments as they do not represent a true scenario of dissociation from a solid tissue.

Clark et al. recently reported cell frequency estimates based on quantitative renal anatomy. However, these were restricted to renal epithelial cells [34]. Based on these estimates, some cell types, such as podocytes, appear to be better represented in scRNA-seq, whereas others, such as loop of Henle cells, were captured more effectively by snRNA-seq. We also attempted to use computational deconvolution of bulk RNA-seq of intact kidneys to infer its cell composition. However, the approach is sensitive to the input marker gene list used and may overlook rare and novel cell types. In addition, cell abundance estimates from bulk data would be influenced by both cell number and relative mRNA content of each cell. We will continue to search for approaches to better define the “ground truth” for cell composition.

In this study, we reported on a range of biases of experimental procedures. We would like to stress that computational tools are being actively developed to mitigate these biases. These include tools for decontamination such as DecontX [38] or SoupX [39], tools for batch-effect removal such as Seurat [30] and Harmony [40], and many others. Hence, even when a dataset in hand is affected by pronounced technical artifacts, certain computational techniques might be useful in reducing their effect.

## Conclusions

From our experiments, we have confirmed several observations by others in the field that have direct relevance to designing single-cell experiments studying human disease biology. Specifically, the depletion of epithelial cell types in cryopreserved samples is concerning given the majority of human cancers are epithelial in origin. Similarly, the underrepresentation of T, B, and NK cells in snRNA-seq data is of critical relevance for studies of tumor immunology, tumor immunotherapy, and autoimmune disease. By comparing these protocols across a single system (mouse kidney), we are now using these results to guide our collaborative network on the best approach for their circumstances. We are instructing our collaborators to use cold dissociation and freshly profile samples wherever possible. For clinical samples (where we often need to wait for pathology results, or are constrained by how archival material has been stored), we discuss with the collaborators the different biases of cryopreservation, methanol fixation, or snRNA-seq from frozen tissue and together decide the best approach. Where information on lymphocytes (T, B, NK) is required, we do not currently recommend snRNA-seq. Researchers will undoubtedly continue to develop new methods aimed to reduce bias. Given the high cost of these experiments, it is critical that these methods are systematically compared to allow the community to decide which to adopt.

## Methods

### Mice

Acknowledging the principles of 3Rs (Replacement, Reduction, and Refinement), all kidneys used in this study were from mice that were euthanized by cervical dislocation as parts of other ongoing ethically approved experiments. In the first series of experiments, comparing cold and warm tissue dissociation and two preservation protocols, male AFAPIL.1DEL C57BL/6J mice from the same litter were used. These mice were 19 weeks old when euthanized and had no exposure to any experimental procedures. For the subsequent experiments, comparing cold-dissociated scRNA-seq to single-nuclei isolation protocols, we used untreated 18-week-old male Balb/c mice from the same litter or untreated 15-week-old female wild type Balb/c mice that were previously used as breeders, as specified in Additional file 17.

### Kidney harvesting

Mice were euthanized and their kidneys were dissected and placed into a 1.5-mL tube containing 1 mL of ice-cold PBS. The capsules were then removed on ice, and the samples processed as detailed below.

### Warm tissue dissociation

Kidneys were dissociated using the Multi-tissue dissociation kit 2 from Miltenyi Biotec [130-110-203] as per manufacturers’ instruction, with minor variations. Once the weight of the kidney was determined, the kidney was quartered and placed into a gentleMACS C-tube [Miltenyi Biotech; 130-096-334] containing the enzyme mix described in the kit’s protocol. The tube was centrifuged briefly, then placed onto the gentleMACS octo dissociator (Miltenyi Biotech), and the 37C_Multi_E program was run after attaching the heating elements. Following completion of the program, the tube was briefly centrifuged. Complete tissue dissociation was checked by examining under the microscope and confirming the absence of visible tissue chunks.

The homogenate was filtered through a 70-μm cell strainer [Greiner; 54,207] into a 50-mL centrifuge tube [Greiner; 227,270], the strainer was then rinsed with 15 mL of PBS. The cell suspension was centrifuged at 400*g* for 10 min; once complete, the supernatant was removed and the pellet was resuspended in 5 mL of PBS + 0.04% BSA [Sigma; A7638]. The cell suspension was then filtered through a 40-μm strainer [Greiner; 542,040], which was subsequently rinsed with 2 mL of PBS + 0.04% BSA. The cells were again centrifuged at 400*g* for 10 min. The supernatant was then removed, and the pellet was resuspended in 5 mL of PBS + 0.04% BSA. Cell count and viability was estimated using the Countess II FL (Thermo Fisher) and the ReadyProbes Blue/Red kit [Invitrogen; R37610]. The cells were then diluted to 700 cells/μL and were immediately loaded onto a 10x chip A and processed on the 10x Chromium controller. The remaining cells were then either methanol fixed or cryopreserved. Cell viability is available in Additional file 18.

### Cold tissue dissociation

Kidneys were dissociated using a modified version of the published protocol described in [18]. Based on the weight, in a pre-cooled Miltenyi C-tube, a protease solution (5 mM CaCl_2_ [Invitrogen; AM9530G], 10 mg/mL *B. Licheniformis* protease [Sigma; P5380], 125 U/mL DNase I [Sigma; D5025], 1xDPBS) was prepared for each kidney.

The kidneys were then minced on ice into a smooth paste using a scalpel. The minced kidney was transferred into 4–6 mL of the protease solution (dependent on weight) and triturated using a 1 mL pipette for 15 s every 2 min for a total of 8 min.

Following trituration, the C-tubes were placed onto a Miltenyi gentleMACS octo dissociator in a cool room (4 °C), and the m_brain_03 program was run twice in succession. Once complete, the samples were triturated for 15 s every 2 min on ice for an additional 16 min using a 1-mL pipette. A total of 10 μL of each sample was then loaded into a hemocytometer to assess whether tissue dissociation was complete. Complete tissue dissociation was also checked by examining under the microscope and confirming the absence of visible tissue chunks. The dissociated cells were transferred to a 15-mL centrifuge tube and 3 mL of ice-cold PBS + 10%FBS [Gibco; A3160401] was added.

The cell suspension was centrifuged at 1200*g* for 5 min at 4 °C. The supernatant was removed and the pellet was resuspended in 2 mL of PBS + 10%FBS. The cells were then filtered through a 70-μm cell strainer, which was subsequently rinsed with 2 mL of PBS + 0.01% BSA. The cells were then centrifuged again at 1200*g* for 5 min at 4 °C followed by removal of the supernatant and resuspension of the pellet in 5 mL of PBS + 0.01%BSA. The cells were then filtered through a 40-μm cell strainer, which was subsequently rinsed with 2 mL of PBS + 0.01% BSA. The cells were again centrifuged at 1200*g* for 5 min at 4 °C followed by removal of the supernatant and resuspension of the cells in 5 mL of PBS + 0.04%BSA. The cells were counted and checked for viability using the ReadyProbes Blue/Red Kit on the Countess II FL. The cells were further diluted to a concentration of 700 cells/μL with PBS/0.04%BSA and loaded directly onto a 10x chip (A/B depending on experiment) and isolated using the 10x Chromium controller. The remaining cells were either methanol fixed or cryopreserved. Cell viability is available in Additional file 18.

### Methanol fixation

#### Fixing

The methanol-fixation protocol was based on [41]. After tissue dissociation, the cells were concentrated to approximately 5 × 10^6^ cells/mL by centrifuging at 1000*g* for 10 min. In total, 200 μL of the cell suspensions was aliquoted into 2-mL cryovials resting on ice. A total of 800 μL of 100% methanol [Sigma; 494,437] (chilled at − 20 °C) was then added dropwise to each sample while gently stirring the cells to prevent clumping. The cryovials were stored at − 20 °C for 30 min, then directly transferred to − 80 °C (no gradient cooling).

#### Rehydrating

Cryovials of methanol-fixed cells were removed from − 80 °C and placed on ice to equilibrate to 4 °C (approximately 10 min). The cells were then transferred to a 1.5-mL centrifuge tube and centrifuged at 1000*g* for 5 min at 4 °C. The supernatant was discarded and the pellet was resuspended in a small volume of SSC cocktail (3xSSC [Sigma; S0902], 0.04% BSA, 40 mM DTT [Sigma; 43816], 0.5 U/mL RNasin plus [Promega; N2615]) to reach a concentration of approximately 2000 cells/μL. The cells were then filtered through a pre-wetted (with 1 mL of nuclease-free water) 40-μm pluristrainer mini filter [PluriSelect; 43-10040]. The cells were counted using the ReadyProbes Blue/Red Kit on the Countess II, then adjusted to 2000 cells/μL based on the count. The cells were loaded onto a 10x chip (A/B depending on version used) at a volume that dilutes the SSC to 0.125x to prevent reverse transcription inhibition. Cell viability is available in Additional file 18.

### Cryopreservation

#### Freezing

After tissue dissociation, the cells were centrifuged at 400*g* for 10 min (1200*g* for 5 min at 4 °C for the repeated experiment), then resuspended in freezing media (50% FBS, 40% RPMI-1640 [Gibco; 11875093], 10% DMSO [Sigma; D4540]) to achieve a concentration of 1 × 10^6^ cells/mL. One milliliter of the cell suspension was aliquoted into 2-mL cryovials, then placed into an isopropanol freezing container (Mr. Frosty) and stored at − 80 °C overnight. The following day, the cells were transferred to liquid nitrogen storage.

#### Thawing

The samples were removed from − 80 °C and immediately placed into a 37 °C waterbath for 2–3 min to rapidly thaw. The cells were then mixed using a 1 mL pipette with a wide-bore tip, and the entire volume was transferred to a 15-mL centrifuge tube [Greiner; 188261]. The cryovial was then rinsed twice with RMPI+ 10%FBS (rinse media); each time, the 1 mL of media was added to the 15-mL centrifuge in a dropwise manner while gently shaking the tube. Seven milliliters of rinse media was added to the centrifuge tube using a serological pipette—the first 4 mL was added dropwise while gently shaking the tube, and the following 3 mL added down the side of the tube over 2 s. The tube was then inverted to mix.

The cells were centrifuged at 300*g* for 5 min. Once completed, the supernatant was removed (leaving 1 mL), placed into another 15-mL centrifuge tube and centrifuged at 400*g* for 5 min. The supernatant was discarded (leaving 1 mL). The pellet from the supernatant was then resuspended, combined with the pellet in the initial centrifuge tube, and mixed. Two milliliters of PBS + 0.04% BSA was added to the centrifuge tube and shaken gently to mix. The cells were then centrifuged again at 400*g* for 5 min. The supernatant was discarded leaving 0.5 mL behind. 0.5 mL of PBS + 0.04% BSA was added to the cells and gently pipette-mixed 10–15 times to fully resuspend. The cells were then filtered through a pre-wetted (with 1 mL of PBS + 0.04% BSA) 40-μm pluristrainer mini filter. A 20 μL aliquot of the cells was used to obtain an estimate of cell count and viability using the ReadyProbes Blue/Red Kit on the Countess II FL. Based on the count, the cells were diluted to a concentration of 700 cells/μL. The cells were then loaded onto a 10x chip (A/B depending on version) and immediately processed on the 10x Chromium controller.

For the repeated experiment, the above method was altered: Rather than a 300*g* spin followed by two 400*g* spins, two 1200*g* spins were performed, omitting the second centrifugation step. Cell viability is available in Additional file 18.

### Flash freezing of whole kidney

Following the removal of the renal capsule, the kidney was placed into an isopentane [Sigma; 320404] bath resting on dry ice for 5 min. The temperature of the bath was maintained between − 30 °C and − 40 °C. Once frozen, the kidney was placed into a pre-cooled (on dry ice) cryovial and then buried in dry ice. The process was repeated for all designated kidneys. The flash-frozen kidneys were then transferred to a − 80 °C freezer for storage.

### Single-nuclei isolation

#### SN_FANS_3x500g

This method is an adaptation of the Frankenstein protocol [42] and the 10x demonstrated protocol [43].

The kidneys were removed from − 80 °C and immediately placed on ice. Each kidney was then transferred to a 1.5-mL tube containing 300 μL of chilled lysis buffer (10 mM Tris-HCl [Invitrogen; AM9856], 3 mM MgCl_2_ [Invitrogen; AM9530G], 10 mM NaCl [Sigma; 71386], 0.005% Nonidet P40 substitute [Roche; 11754599001], 0.2 U/mL RNasin plus) and incubated on ice for 2 min. The tissue was then completely homogenized using a pellet pestle [Fisherbrand; FSB12-141-364] using up and down strokes without twisting. 1.2 mL of chilled lysis buffer was added to the tube and pipette-mixed (wide-bore). The full volume was then transferred to a pre-cooled 2-mL tube. The homogenate was incubated on ice for 5 min and mixed with a wide-bore tip every 1.5 min.

Following the incubation, 500 μL of the lysis buffer was added to the homogenate, which was subsequently pipette-mixed and split equally into four 2-mL tubes. One milliliter of chilled lysis buffer was added to each tube and pipette-mixed using a wide-bore tip. The four tubes were incubated for a further 5 min on ice, mixing with a wide-bore tip every 1.5 min. The homogenate from the four tubes was then filtered through a 40-μm strainer into a pre-cooled 50-ml centrifuge tube. Following this, the sample was split again into four 2-mL tubes resting on ice.

The samples were centrifuged at 500*g* for 5 min at 4 °C. The supernatant was removed leaving 50 μL in the tube. 1.5 mL of lysis buffer was then added to two of the tubes and the pellet resuspended by mixing with a pipette. This resulted in two tubes containing 1.5 mL resuspended nuclei in lysis buffer, and two tubes containing a nuclei pellet in 50 μL of lysis buffer. The resuspended nuclei in one tube was then combined with the nuclei pellet of another, resulting in two tubes containing resuspended nuclei in lysis buffer.

The nuclei were centrifuged again at 500*g* for 5 min at 4 °C. The supernatant was removed completely and discarded. A total of 500 μL of nuclei wash buffer (1xDPBS, 1% BSA, 0.2 U/mL RNasin plus) was added to the tube containing the pellet and left to incubate without resuspending for 5 min. Following incubation, an additional 1 mL of nuclei wash buffer was added, and the nuclei were resuspended by gently mixing with a pipette. The nuclei were again centrifuged at 500*g* for 5 min at 4 °C, followed by discarding the supernatant. The pellets were resuspended in 1.4 mL of nuclei wash buffer, then transferred into a pre-cooled 1.5-mL tube. Another 500*g* centrifugation step for 5 min at 4 °C was performed. The supernatant was then discarded, and the nuclei pellet was resuspended in 1 mL of nuclei wash buffer.

The nuclei were then filtered through a 4-μm pluristrainer mini filter. A total of 200 μL of the filtered nuclei suspension was transferred into a 0.5-mL tube and set aside to be used as the unstained control for sorting. To the remaining 800 μL, 8 μL of DAPI (10 μg/mL) [Thermo Scientific; 62248] was added, and the nuclei were mixed with a pipette. A quality control step was performed by viewing the nuclei under a fluorescence microscope on a hemocytometer to check nuclei shape and count.

A BD Influx Cell Sorter was then used to sort 100,000 DAPI-positive events using a 70-μm nozzle and a pressure of 22 psi (as per gating strategy, Additional file 2: Figure S10). The post-sort nuclei concentration and quality were then checked using a fluorescence microscope and hemocytometer. Nuclei were then loaded onto a 10x chip (A/B depending on version used) and processed immediately on the 10x Chromium controller.

#### SN_FANS_1x2000g

The flash-frozen kidneys were removed from − 80 °C and transferred to a 1.5-mL tube containing 500 μL of pre-chilled lysis buffer same recipe as previous protocol) and allowed to rest on ice for 2 min. Each kidney was then homogenized with a pellet pestle with 40 up and down strokes without twisting the pellet. The resulting homogenate was mixed with a pipette and transferred to pre-cooled 15-mL centrifuge tube containing 2 mL of lysis buffer. The homogenate was incubated for 12 min on ice with mixing every 2 min using a glass fire-polished silanized Pasteur pipette [Kimble; 63A54]. Once incubation was complete, 2.5 mL of nuclei wash buffer (same recipe as previous protocol) was added to the homogenate. The remaining tissue fragments were completely dissociated by repeated trituration of the homogenate using the glass Pasteur pipette.

The homogenate was then filtered through a 30-μm MACS Smart Strainer [Miltenyi Biotech; 130-098-458] into a new 15-mL centrifuge tube. The nuclei were centrifuged at 2000*g* for 5 min at 4 °C. The supernatant was removed and the nuclei pellet was resuspended in 1 mL of nuclei wash buffer. A total of 200 μL was aliquoted into a 0.5-mL tube to be used as an unstained control for sorting. Eight microliters of DAPI (10 μg/mL) was added to the remaining 800 μL of nuclei. Quality and quantity of the nuclei was checked using a fluorescence microscope prior to sorting. Sorting and post sorting QC was performed in the same manner as for the SN_FANS_3x500g protocol. Nuclei were then loaded onto a 10x chip B and processed immediately on the 10x Chromium controller.

#### SN_sucrose

Kidneys were removed from − 80 °C and transferred to a 1.5-mL tube containing 500 μL of pre-chilled lysis buffer II (same recipe as previous protocols, with 125 U/mL of DNase I added) and allowed to rest on ice for 2 min. Each kidney was then homogenized using a pellet pestle with 40 up and down strokes without twisting. The homogenate was transferred to a 15-mL centrifuge tube containing 2 mL of lysis buffer II and incubated for 12 min on ice with mixing every 2 min using a glass fire-polished silanized Pasteur pipette. Following the incubation, 2.5 mL of nuclei wash buffer II (1× DPBS + 2%BSA) was added to the homogenate. Remaining tissue clumps were dissociated by repeated trituration of the homogenate using the glass Pasteur pipette.

The homogenate was then filtered through a 30-μm MACS Smart Strainer into a new 15-mL centrifuge tube. Subsequently, the homogenate was centrifuged at 2000*g* for 5 min at 4 °C. The supernatant was removed, and the pellet was resuspended in 510 μL of nuclei wash buffer II. Ten microliters of the suspension was transferred to a 1.5-mL tube and placed on ice for use in nuclei recovery calculations. A total of 900 μL of 1.8 M sucrose solution [Sigma; NUC201] was added to the remaining 500 μL of nuclei suspension and homogenized by mixing with a pipette. 3.6 mL of 1.3 M sucrose solution [Sigma; NUC201] was added to a 5-mL tube. The nuclei/sucrose homogenate was then gently layered on top of the 1.3 M sucrose solution.

The 5-mL tube containing the sucrose solutions and nuclei was then centrifuged at 3000*g* for 10 min at 4 °C. Once centrifugation was complete, the sucrose phase containing debris was soaked up using a Kimwipe wrapped around a pellet pestle. The remaining supernatant was removed and discarded using a pipette. The nuclei pellet was then resuspended in 5 mL of wash buffer II, of which 10 μL was transferred to a 1.5-mL tube to assess nuclei recovery.

To the 10 μL of nuclei suspension removed prior to the sucrose gradient, 980 μL of wash buffer II and 10 μL of DAPI (10 μg/mL) was added. To the 10 μL of nuclei suspension removed after the sucrose gradient, 89 μL of wash buffer II and 1 μL of DAPI (10 μg/mL) was added. The yield from the pre- and post-sucrose aliquots was compared to assess nuclei recovery after filtration through the gradient. The post-sucrose count was used to dilute the nuclei to a concentration of 700 nuclei/μL, which was immediately loaded onto a 10x chip B and processed with the 10x Chromium controller.

### Single-cell RNA-seq library preparation

All single-cell libraries were constructed in biological triplicate using the 10x Chromium 3′ workflow as per the manufacturers’ directions. In the first series of experiments, comparing cold and warm tissue dissociation and two preservation protocols, version 2 chemistry was used. For single-cell versus single-nuclei comparisons, versions 2 and 3 were used as indicated in Additional file 17. All experiments and conditions aimed for a capture of approximately 9000 cells, except for methanol-fixed samples. Due to the reverse transcription inhibition of 3x SSC, the sample had to be loaded at a concentration of 0.125x SSC, resulting in an approximate cell capture of 4000–5000 cells.

### Bulk RNA-seq library preparation

For the undissociated samples, total RNA was extracted from flash-frozen kidneys using the Nucleospin RNA Midi kit [Macherey Nagel; 740,962.20] as per the manufacturers’ directions. For the dissociated samples, total RNA was extracted from the remaining cells from each of the tissue dissociation protocols. RNA was assessed for quantity and quality using the TapeStation 4200 RNA ScreenTape kit [Agilent; 5067-5576], which showed all RNA used had a RIN of > 8. Bulk RNA-seq was performed using the NEBNext Ultra II RNA Library Kit for Illumina [NEB; E7760] and either NEBNext rRNA Depletion Kit (Human/Mouse/Rat) [NEB; E6310] or NEBNext Poly(A) mRNA isolation module [NEB; E7490] as described in the manufacturers’ protocol, with 100 ng of total RNA as input.

### Sequencing

All libraries were quantified with qPCR using the NEBnext Library Quant Kit for Illumina and checked for fragment size using the TapeStation D1000 kit (Agilent). The libraries were pooled in equimolar concentration for a total pooled concentration of 2 nM. 10x single-cell libraries were sequenced using the Illumina NovaSeq 6000 and S2 flow cells (100 cycle kit) with a read one length of 26 cycles, and a read two length of 92 or 98 cycles for version 2 chemistry. Version 3 chemistry had a read one length of 28 cycles, and a read two length of 94 cycles. Bulk libraries were sequenced on the Illumina NovaSeq 6000 using SP flow cells (100 cycle kit) with read length of 150 for dissociated bulk in C57BL/6J mice, 51 for undissociated bulk in Balb/c male mice, and 60 for Balb/c female mice.

### Bulk RNA-seq data processing

BCL files were demultiplexed and converted into FASTQ using bcl2fastq utility of Illumina BaseSpace Sequence Hub. FastQC was used for read quality control [44]. Adapters and low-quality bases were trimmed using Trim Galore with parameters *--paired --quality 5 --stringency 5 --length 20 --max_n 10* [45]. Reads matching to ribosomal DNA repeat sequence BK000964 [46] and low complexity reads were removed with TagDust2 [47]. The remaining reads were mapped to GRCm38.84 version of mouse genome using STAR version 2.6.1a with default settings [48]. Picard MarkDuplicates tool was employed to identify duplicates [49]. FeatureCounts was then used to derive gene count matrix [50]. Counts were normalized to gene length and then to library sizes using weighted trimmed mean of M-values (TMM) method in edgeR [27], to derive gene length corrected trimmed mean of M-values (GeTMM) as described in [29].

### scRNA-seq and snRNA-seq data processing

BCL files were demultiplexed and converted into FASTQ using bcl2fastq utility of Illumina BaseSpace Sequence Hub. scRNA-seq and snRNA-seq libraries were processed using Cell Ranger 2.1.1 with mm10-2.1.0 reference. Reads mapped to exons were used for scRNA-seq samples, whereas both intronic and exonic reads were counted for snRNA-seq. Custom pre-mRNA reference for snRNA-seq was built as described in [51]. Raw gene-barcode matrices from Cell Ranger output were used for downstream processing. Cells were distinguished from background noise using EmptyDrops [52]. Only genes detected in a minimum of 10 cells were retained; cells with 200–3000 genes and under 50% of mitochondrial reads were retained, as per Park et al. study [32]. Nuclei were additionally filtered to have at least 450 UMIs for v2 chemistry and 900 UMIs for v3 chemistry, and mitochondrial genes were removed. Outlier cells with high ratio of number of detected UMI to genes (> 3 median absolute deviations from median) were removed using Scater [53]. Seurat v2 was used for sample integration (canonical correlation analysis), normalization (dividing by the total counts, multiplying by 10,000 and natural-log transforming), scaling, clustering, and differential expression analysis (Wilcoxon test) [30].

### Inferring cell identity

To infer cell identity for freshly profiled samples in the first series of experiments, we performed a reference-based annotation using scMatch [31] and refined cell labels based on marker gene expression in a two-step procedure described below (Additional file 2: Figure S1).

#### Reference dataset

To construct the reference dataset for scMatch [31], we obtained gene counts and cell types reported in three single-cell (or single-nuclei) adult mouse kidney studies [26, 32, 33]. Counts were normalized to cell library size and averaged within each cell type to derive reference vectors (Additional file 2: Figure S1, Step 1). The reference vectors were clustered using Spearman correlation coefficient, and five vectors were removed as outliers. The remaining 66 vectors composed a reference dataset, available as Additional file 19. With this reference dataset, we ran scMatch [31] (Additional file 2: Figure S1, Step 2) using options *--testMethod s --keepZeros y* to label each individual cell with the closest cell type identity from the reference dataset.

#### Refining cell identities

We next refined scMatch-derived cell types based on gene expression. First, for each cell type, we calculated gene signatures as genes overexpressed in the given cell type when compared to all other cells (*FindMarkers* function of Seurat [30], minimum detection rate of 0.5, logFC threshold of 1 and FDR < 0.05 were used as thresholds; only cell types with at least 10 cells were considered; Additional file 2: Figure S1, Step 3). Second, cell type gene signature scores were calculated for each cell and for each gene signature (*AddModuleScore* function of Seurat [30], genes attributed to signatures in more than two cell types were excluded; Additional file 2: Figure S1, Step 4). Third, we used these scores to assign cell types to cells (Additional file 2: Figure S1, Step 5). A cell type was assigned to a cell if the score for that cell type was the highest among all cell types, positive and significant with FDR < 0.05. Significance was determined in a Monte-Carlo procedure with 1000 randomly selected gene sets of the same size [54], correction for multiple hypothesis testing was performed using Benjamini-Hochberg procedure [55]. Cells without cell type annotation were manually explored to identify whether the corresponding cell type might be a novel one, absent from the reference.

#### Second iteration

Cell types inferred in our dataset were added to the reference dataset (Additional file 2: Figure S1, Step 6), and annotation with scMatch and gene set signature scoring was repeated. Cells left unannotated at this stage were labelled as “unknown.” Cell type gene signatures are available in Additional file 20.

This approach failed to identify cells of connecting tubule (CNT) and, instead, matched them to other similar cell types. To resolve this, annotation for cell types labelled as DCT, aLOH, CD_IC, CD_PC, and CD_Trans was additionally refined as follows. These cells were extracted from the dataset and clustered separately. Candidate CNT cells were identified as a cluster overexpressing *Calb1* and *Klk1* genes [34, 56]. The cell type signature score procedure was then applied for this subset as described above.

Cell type labels assigned to each cell are available in Additional file 3.

#### Preserved cells

Cells of preserved single-cell suspensions from the first series of experiments were annotated using the cell type gene signatures derived from the corresponding freshly profiled samples (Additional file 20) and the gene set signature scoring procedure described above. Cell type labels assigned to each cell are available in Additional file 3.

#### Subsequent experiments

In subsequent experiments, we used a combined reference dataset, which included the public data as well as data from freshly profiled cells generated in the first series of experiments (Additional file 21, note that two cell types were excluded from the reference as outliers). Single-cell datasets were annotated using a single iteration of scMatch. For single-nucleus datasets, we repeated the two-step annotation procedure described above. Cell type labels assigned to each cell or nucleus are available in Additional file 3.

### Stress response score

To select genes for the stress response score, we looked at all genes induced in the warm-dissociated samples, and from that list, we manually selected genes which have been reported in the literature as stress-response-related genes. Stress response score was calculated for 17 genes (*Fosb*, *Fos*, *Jun*, *Junb*, *Jund*, *Atf3*, *Egr1*, *Hspa1a*, *Hspa1b*, *Hsp90ab1*, *Hspa8*, *Hspb1*, *Ier3*, *Ier2*, *Btg1*, *Btg2*, *Dusp1*) for each cell using *AddModuleScore* function of Seurat version 2 [30]. The score represents an average expression level of these genes on a single-cell level, subtracted by the aggregated expression of control gene sets. All analyzed genes were binned based on averaged expression, and the control genes were randomly selected from each bin. Significance was determined in a Monte-Carlo procedure with 1000 randomly selected sets of 17 genes [54], correction for multiple hypothesis testing was performed using Benjamini-Hochberg procedure [55].

### Cell cycle phase prediction

Cell cycle phases were inferred using *CellCycleScoring* function of Seurat version 2 [30] with the following genes: S-genes: *Atad2*, *Blm*, *Brip1*, *Casp8ap2*, *Ccne2*, *Cdc45*, *Cdc6*, *Cdca7*, *Chaf1b*, *Clspn*, *Dscc1*, *Dtl*, *E2f8*, *Exo1*, *Fen1*, *Gins2*, *Gmnn*, *Hells*, *Mcm2*, *Mcm4*, *Mcm5*, *Mcm6*, *Msh2*, *Nasp*, *Pcna*, *Pcna-ps2*, *Pola1*, *Pold3*, *Prim1*, *Rad51ap1*, *Rfc2*, *Rpa2*, *Rrm1*, *Rrm2*, *Slbp*, *Tipin*, *Tyms*, *Ubr7*, *Uhrf1*, *Ung*, *Usp1*, *Wdr76*; G2M-genes: *Anln*, *Anp32e*, *Aurka*, *Aurkb*, *Birc5*, *Bub1*, *Cbx5*, *Ccnb2*, *Cdc20*, *Cdc25c*, *Cdca2*, *Cdca3*, *Cdca8*, *Cdk1*, *Cenpa*, *Cenpe*, *Cenpf*, *Ckap2*, *Ckap2l*, *Ckap5*, *Cks1brt*, *Cks2*, *Ctcf*, *Dlgap5*, *Ect2*, *G2e3*, *Gas2l3*, *Gtse1*, *Hjurp*, *Hmgb2*, *Hmmr*, *Kif11*, *Kif20b*, *Kif23*, *Kif2c*, *Lbr*, *Mki67*, *Ncapd2*, *Ndc80*, *Nek2*, *Nuf2*, *Nusap1*, *Psrc1*, *Rangap1*, *Smc4*, *Tacc3*, *Tmpo*, *Top2a*, *Tpx2*, *Ttk*, *Tubb4b*, *Ube2c*. Note cells not annotated as S or G2M phase are by default labelled as G1 phase.

### Bulk RNA-seq deconvolution

BSEQ-sc was used for bulk expression deconvolution [35]. In the first series of experiments, marker genes for the deconvolution were calculated from scRNA-seq data, using only cold-dissociated samples to avoid the influence of the identified warm dissociation-related biases. We also excluded cells labelled as “Unknown” and “CD_Trans” from the calculation. For each of the remaining cell types, marker genes were calculated using Seurat function *FindMarkers* with the following thresholds: *logfc.threshold = 1.5*, *min.pct = 0.5*, *only.pos = T*. Genes identified in more than one cell type were removed, and the remaining genes were used for the deconvolution. The same set of genes was used to deconvolve all bulk RNA-seq libraries.

## Supplementary information

**Additional file 1: Table S1.** Genes differentially expressed between bulk RNA-seq profiles of cold- and warm-dissociated kidney single-cell suspensions (FDR < 0.05 and logFC > 2, edgeR exact test [27]); includes results of functional analysis with ToppGene [28] and Gene Ontology Biological Process for differentially expressed genes with higher expression in warm-dissociated kidneys.

**Additional file 2: Supplementary Figures. Figure S1.** Cell annotation procedure. **Figure S2.** TSNE plots showing freshly profiled cells from cold- and warm-dissociated kidneys. **Figure S3.** Expression and detection levels of selected marker genes. **Figure S4.** Cell preservation protocol performance in warm-dissociated samples. **Figure S5.** Cell type composition of fresh and preserved kidneys. **Figure S6.** Cell type composition of non-proximal tubule cells in fresh and preserved kidneys. **Figure S7.** Cell type composition of fresh and cryopreserved cold-dissociated samples in the repeated experiment using Balb/c female mice, 10x v3 chemistry, 2 weeks storage, and 1200 g spin. **Figure S8.** Comparison of single-cell and single-nucleus libraries in Balb/c male mice. **Figure S9.** Cell cycle phases inferred in scRNA-seq and snRNA-seq libraries from Balb/c male mice. **Figure S10.** FANS gating strategy. **Figure S11.** BSEQ-sc deconvolution of bulk RNA-seq profiles of cold- and warm-dissociated kidney single-cell suspensions. Three biological replicates are shown per condition. **Figure S12.** Comparison of ambient RNA contamination in methanol-fixed and freshly profiled aliquots of cold-dissociated samples. **Figures S13–14.** Comparison of nuclei isolation protocols. **Figure S15.** Comparison of bulk RNA-seq profiles of intact kidneys and cold-dissociated single-cell suspensions. **Figure S16**. Expression of genes differentially expressed between bulk RNA-seq profiles of intact and dissociated kidneys in the matching single-cell dataset, Balb/c female mice.

**Additional file 3: Table S2.** Cell type labels assigned to cells and nuclei in this study. aLOH: ascending loop of Henle; CD_IC: intercalated cells of collecting duct; CD_IC_A: type A intercalated cells of collecting duct; CD_IC_B: type B intercalated cells of collecting duct; CD_PC: principal cells of collecting duct; CD_Trans: transitional cells; CNT: connecting tubule; DCT: distal convoluted tubule; dLOH: descending loop of Henle; MC: mesangial cells; MPH: macrophages; PT: proximal tubule.

**Additional file 4: Table S3.** Differentially expressed genes with higher expression in cell populations of warm-dissociated kidneys (Seurat Wilcoxon test [30] with thresholds of logFC = 0.5, minimum detection rate 0.5, FDR < 0.05); includes an incidence table indicating in how many cell types each gene was identified as differentially expressed and results of functional analysis with ToppGene [28] and Gene Ontology Biological Process for differentially expressed genes identified in at least one cell type.

**Additional file 5: Table S4.** Differentially expressed genes with higher expression in cell populations of cold-dissociated kidneys (Seurat Wilcoxon test [30] with thresholds of logFC = 0.5, minimum detection rate 0.5, FDR < 0.05); includes an incidence table indicating in how many cell types each gene was identified as differentially expressed.

**Additional file 6.** Supplementary Notes.

**Additional file 7: Table S5.** Genes differentially expressed between cryopreserved and freshly profiled cold-dissociated kidney single-cell suspensions (Seurat Wilcoxon test [30] with thresholds of logFC = 1, minimum detection rate 0.5, FDR < 0.05), positive logFC indicates higher expression in cryopreserved samples; includes incidence tables indicating in how many cell types each gene was identified as differentially expressed.

**Additional file 8: Table S6.** Genes differentially expressed between cryopreserved and freshly profiled warm-dissociated kidney single-cell suspensions (Seurat Wilcoxon test [30] with thresholds of logFC = 1, minimum detection rate 0.5, FDR < 0.05), positive logFC indicates higher expression in cryopreserved samples; includes incidence tables indicating in how many cell types each gene was identified as differentially expressed.

**Additional file 9: Table S7.** Genes differentially expressed between methanol-fixed and freshly profiled cold-dissociated kidney single-cell suspensions (Seurat Wilcoxon test [30] with thresholds of logFC = 1, minimum detection rate 0.5, FDR < 0.05), positive logFC indicates higher expression in methanol-fixed samples; includes incidence tables indicating in how many cell types each gene was identified as differentially expressed.

**Additional file 10: Table S8.** Genes differentially expressed between methanol-fixed and freshly profiled warm-dissociated kidney single-cell suspensions (Seurat Wilcoxon test [30] with thresholds of logFC = 1, minimum detection rate 0.5, FDR < 0.05), positive logFC indicates higher expression in methanol-fixed samples; includes incidence tables indicating in how many cell types each gene was identified as differentially expressed.

**Additional file 11: Table S9.** Genes differentially expressed in each cell type between SN_FANS_1x2000g and SN_FANS_3x500g protocols (Seurat Wilcoxon test [30] with thresholds of logFC = 0.5, minimum detection rate 0.5, FDR < 0.05), positive logFC indicates higher expression in SN_FANS_1x2000g libraries; includes incidence tables indicating in how many cell types each gene was identified as differentially expressed.

**Additional file 12: Table S10.** Genes differentially expressed in each cell type between SN_FANS_1x2000g and SN_sucrose protocols (Seurat Wilcoxon test [30] with thresholds of logFC = 0.5, minimum detection rate 0.5, FDR < 0.05), positive logFC indicates higher expression in SN_FANS_1x2000g libraries; includes incidence tables indicating in how many cell types each gene was identified as differentially expressed.

**Additional file 13: Table S11.** Genes differentially expressed in each cell type between SN_sucrose and SN_FANS_3x500g protocols (Seurat Wilcoxon test [30] with thresholds of logFC = 0.5, minimum detection rate 0.5, FDR < 0.05), positive logFC indicates higher expression in SN_sucrose libraries; includes incidence tables indicating in how many cell types each gene was identified as differentially expressed.

**Additional file 14: Table S12.** Number of cells in each cell population across single-cell and single-nuclei experiments and BSEQ-sc estimates for bulk RNA-seq libraries.

**Additional file 15: Table S13.** Genes differentially expressed between bulk RNA-seq profiles of intact kidneys and cold-dissociated single-cell suspensions; includes results of functional analysis with ToppGene [28] for genes with higher expression in intact kidneys.

**Additional file 16: Table S14.** Genes differentially expressed between single-cell and single-nuclei libraries in Balb/c male mice profiled with v2 10x chemistry (Seurat Wilcoxon test [30] with thresholds of logFC = 0.5, minimum detection rate 0.5, FDR < 0.05), positive logFC indicates higher expression in single-cell libraries; includes incidence tables indicating in how many cell types each gene was identified as differentially expressed.

**Additional file 17: Table S15.** Mice used and workflows tested. The experiments were performed in three batches separated in time. For each batch, we used kidneys from mice available at that time and the workflows were used with modifications as indicated in this table.

**Additional file 18: Table S16.** Cell viability.

**Additional file 19: Table S17.** Public reference dataset used for the first scMatch run.

**Additional file 20: Table S18.** Cell type gene signatures for refined annotation.

**Additional file 21: Table S19.** Extended reference dataset used for scMatch annotation.

**Additional file 22.** Review history.

## Data Availability

The datasets generated and analyzed during the current study are available from the Gene Expression Omnibus (GEO) repository with the primary accession code GSE141115 [57].
