# Supplementary material for: Systematic assessment of tissue dissociation and storage biases in single-cell and single-nucleus RNA-seq workflows
Source: Genome Biol. 2020 Jun 2;21:130. doi: 10.1186/s13059-020-02048-6 (PMC7265231; doi:10.1186/s13059-020-02048-6)
Supplement: Supplementary file 2 — Additional file 2: Supplementary Figures. Figure S1. Cell annotation procedure. Figure S2. TSNE plots showing freshly profiled cells from cold- and warm-dissociated kidneys. Figure S3. Expression and detection levels of selected marker genes. Figure S4. Cell preservation protocol performance in warm-dissociated samples. Figure S5. Cell type composition of fresh and preserved kidneys. Figure S6. Cell type composition of non-proximal tubule cells in fresh and preserved kidneys. Figure S7. Cell type composition of fresh and cryopreserved cold-dissociated samples in the repeated experiment using Balb/c female mice, 10x v3 chemistry, 2 weeks storage, and 1200 g spin. Figure S8. Comparison of single-cell and single-nucleus libraries in Balb/c male mice. Figure S9. Cell cycle phases inferred in scRNA-seq and snRNA-seq libraries from Balb/c male mice. Figure S10. FANS gating strategy. Figure S11. BSEQ-sc deconvolution of bulk RNA-seq profiles of cold- and warm-dissociated kidney single-cell suspensions. Three biological replicates are shown per condition. Figure S12. Comparison of ambient RNA contamination in methanol-fixed and freshly profiled aliquots of cold-dissociated samples. Figures S13–14. Comparison of nuclei isolation protocols. Figure S15. Comparison of bulk RNA-seq profiles of intact kidneys and cold-dissociated single-cell suspensions. Figure S16. Expression of genes differentially expressed between bulk RNA-seq profiles of intact and dissociated kidneys in the matching single-cell dataset, Balb/c female mice. [file 13059_2020_2048_MOESM2_ESM.pdf]

# Fig. S1

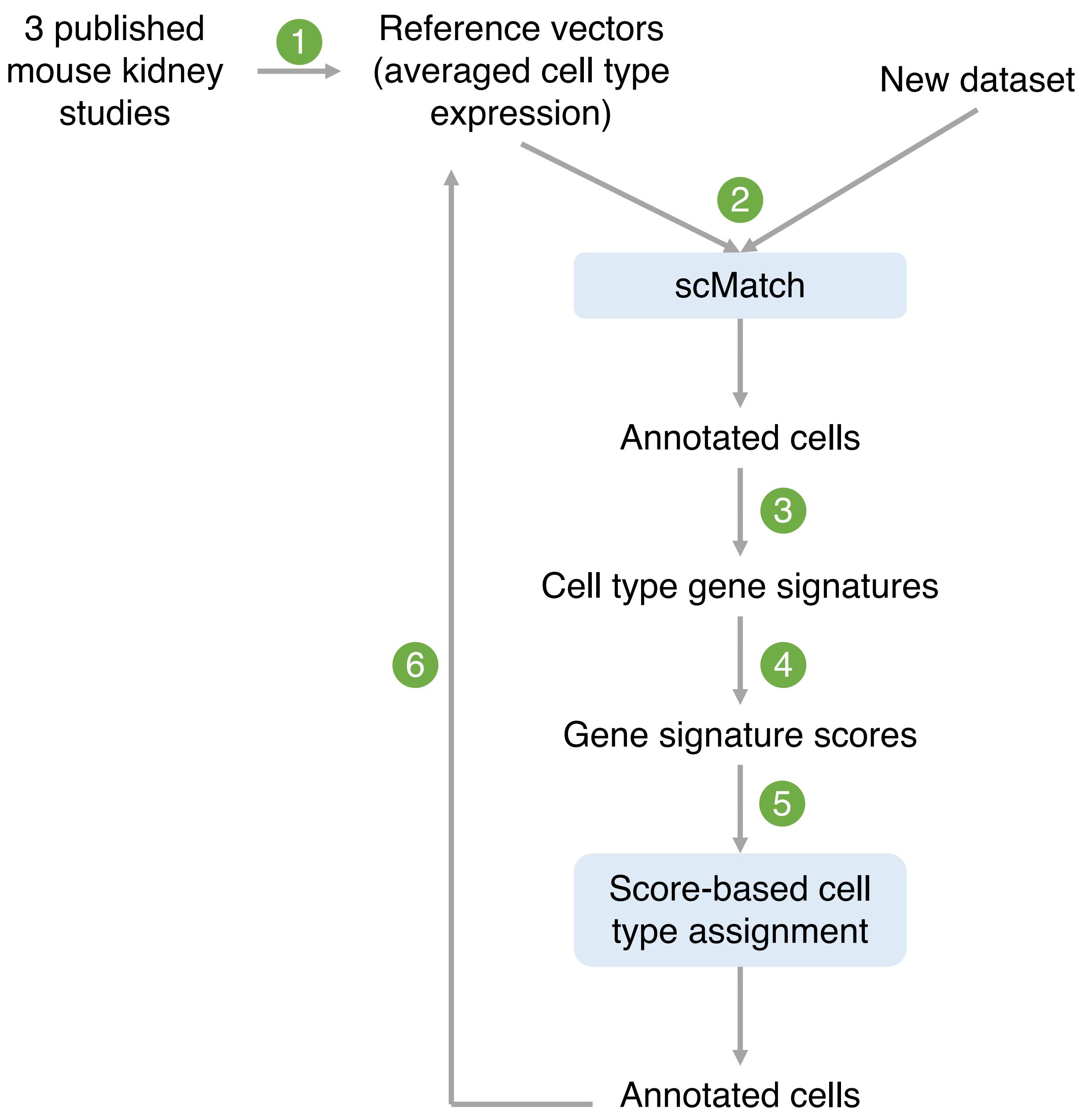


Cell annotation procedure. See **Methods** for more details.

# Fig. S2

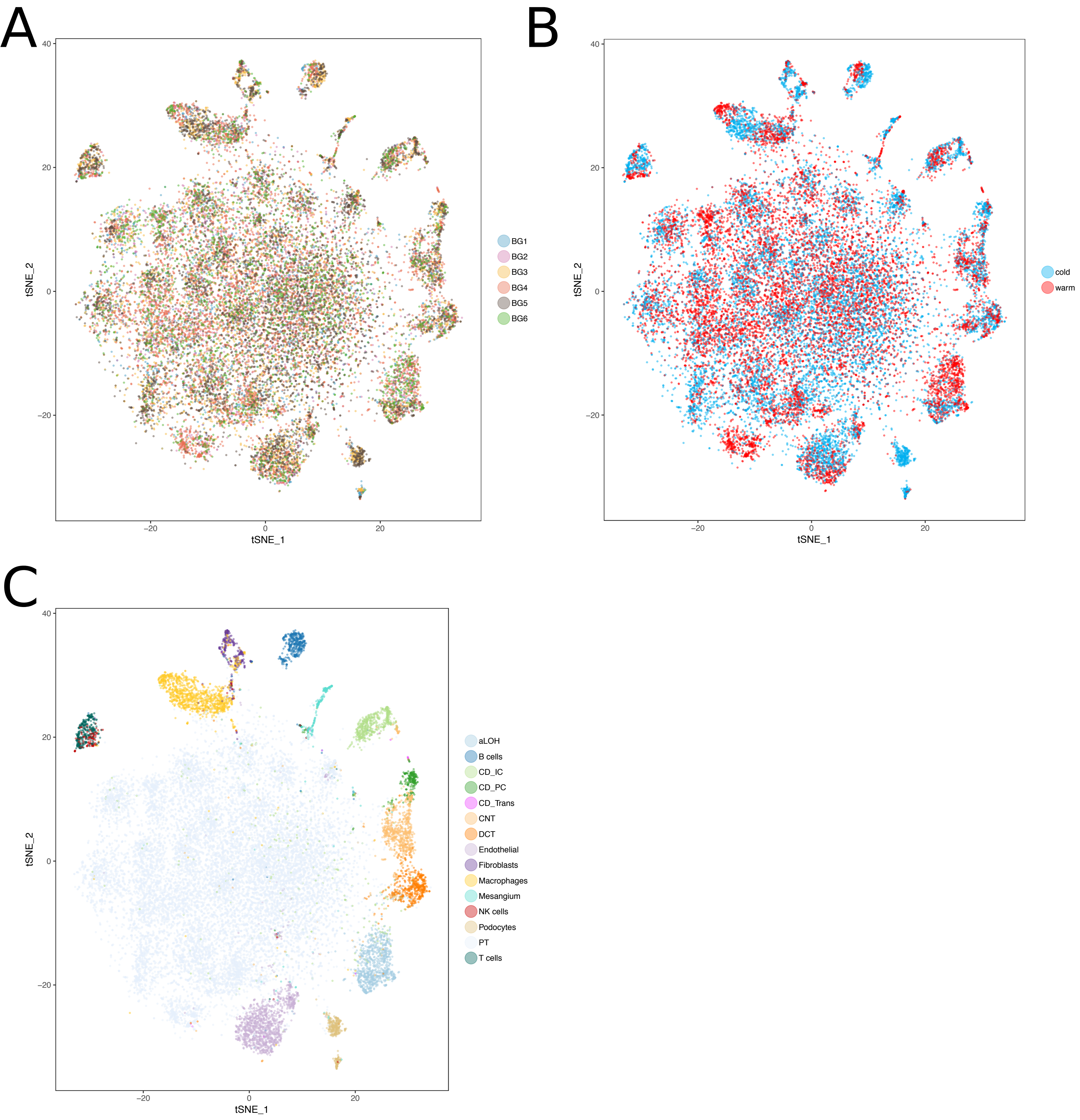


TSNE plots showing freshly profiled cells from cold- and warm-dissociated kidneys coloured by **a**) library, **b**) dissociation protocol, **c**) inferred cell type.

aLOH: ascending loop of Henle; CD_IC: intercalated cells of collecting duct; CD_PC: principal cells of collecting duct; CD_Trans: transitional cells of collecting duct; CNT: connecting tubule; DCT: distal convoluted tubule; PT: proximal tubule.

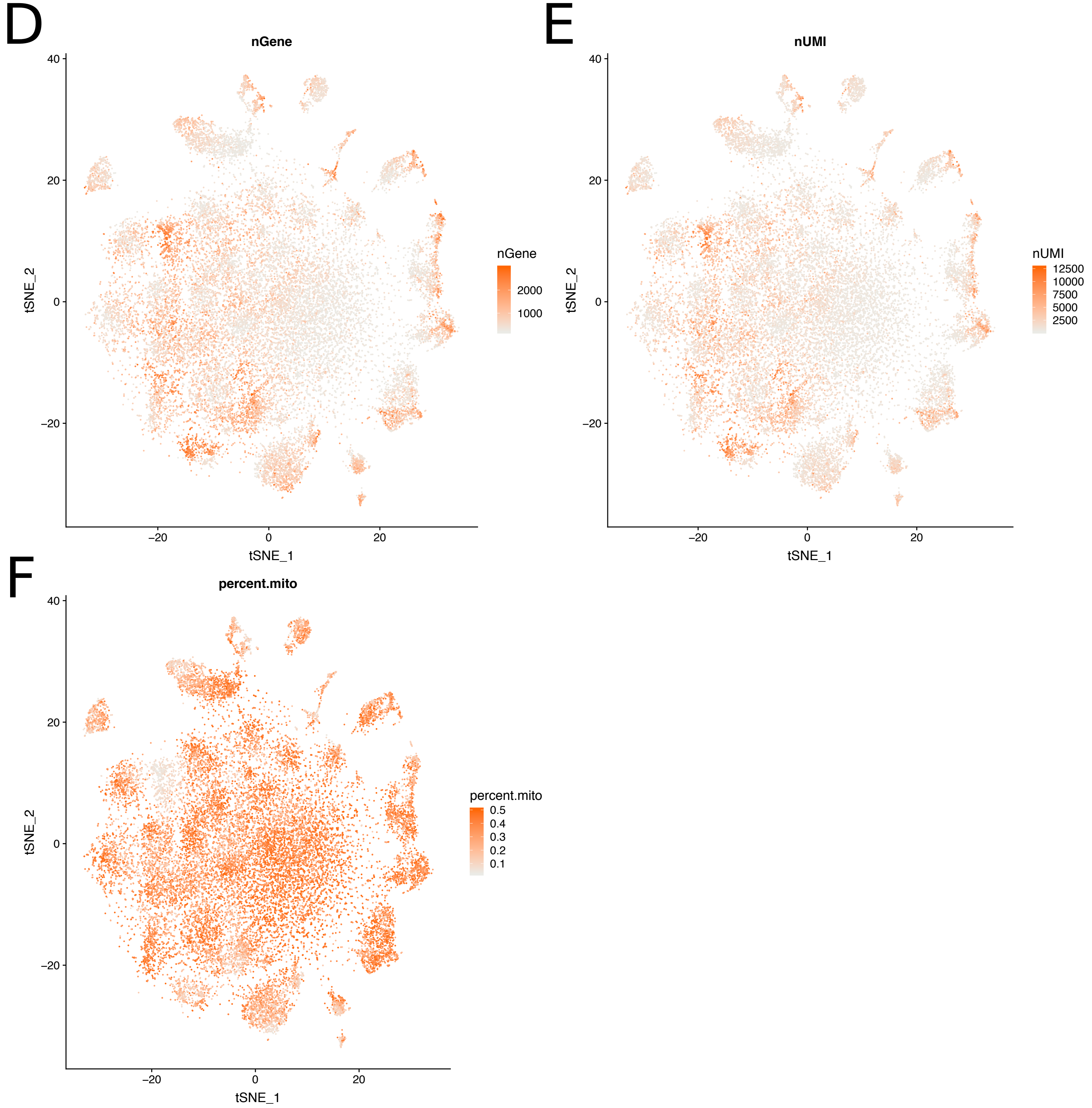


TSNE plots showing freshly profiled cells from cold- and warm-dissociated kidneys coloured by **d**) number of detected genes, **e**) number of UMIs, **f**) fraction of reads mapped to mitochondrial genes.

# Fig. S3

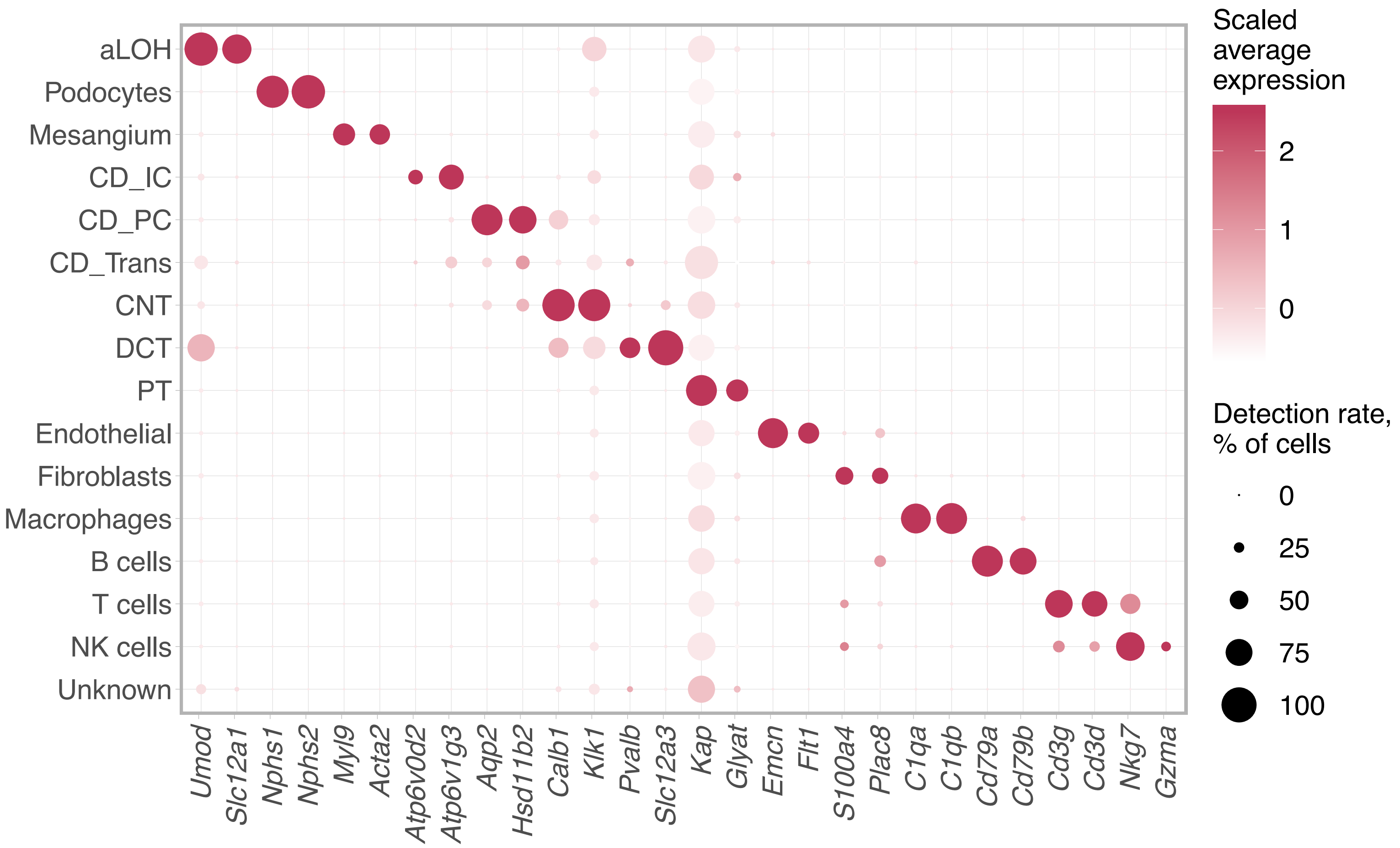


Expression and detection levels of selected marker genes.

aLOH: ascending loop of Henle; CD_IC: intercalated cells of collecting duct; CD_PC: principal cells of collecting duct; CD_Trans: transitional cells of collecting duct; CNT: connecting tubule; DCT: distal convoluted tubule; PT: proximal tubule.

# Fig. S4

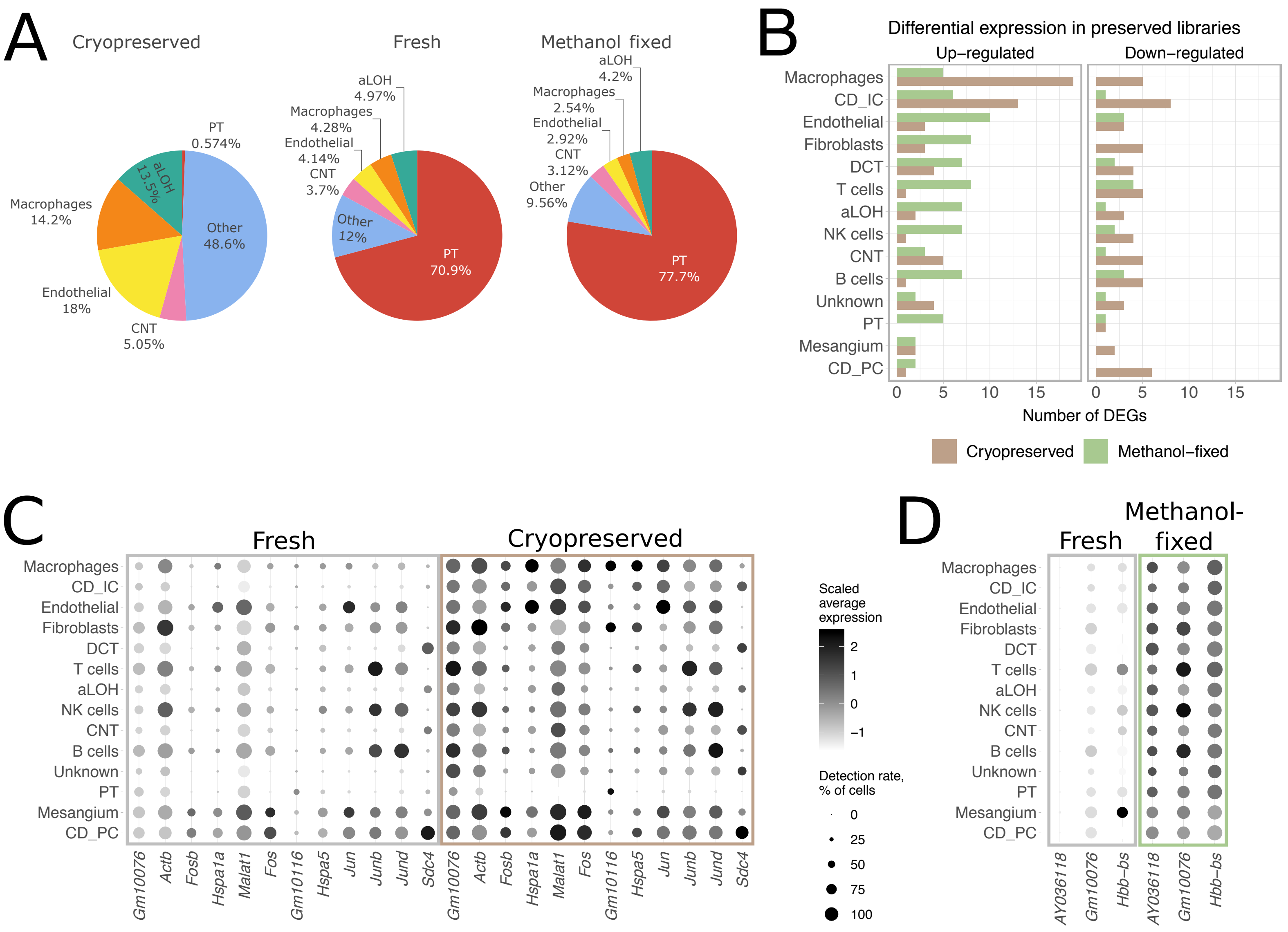


Cell preservation protocol performance in warm-dissociated samples. **a**) Cell type composition of fresh and preserved warm-dissociated samples. **b**) Number of DEGs detected between preserved and freshly profiled aliquots. Seurat Wilcoxon test with logFC = 1, min detection rate 0.5, FDR < 0.05 as thresholds. **c**) Expression and detection rates of DEGs with higher expression in cryopreserved samples in at least two cell types. **d**) Expression and detection rates of DEGs with higher expression in methanol-fixed samples in at least nine cell types.

aLOH: ascending loop of Henle; CD_IC: intercalated cells of collecting duct; CD_PC: principal cells of collecting duct; CD_Trans: transitional cells of collecting duct; CNT: connecting tubule; DCT: distal convoluted tubule; PT: proximal tubule.

# Fig. S5

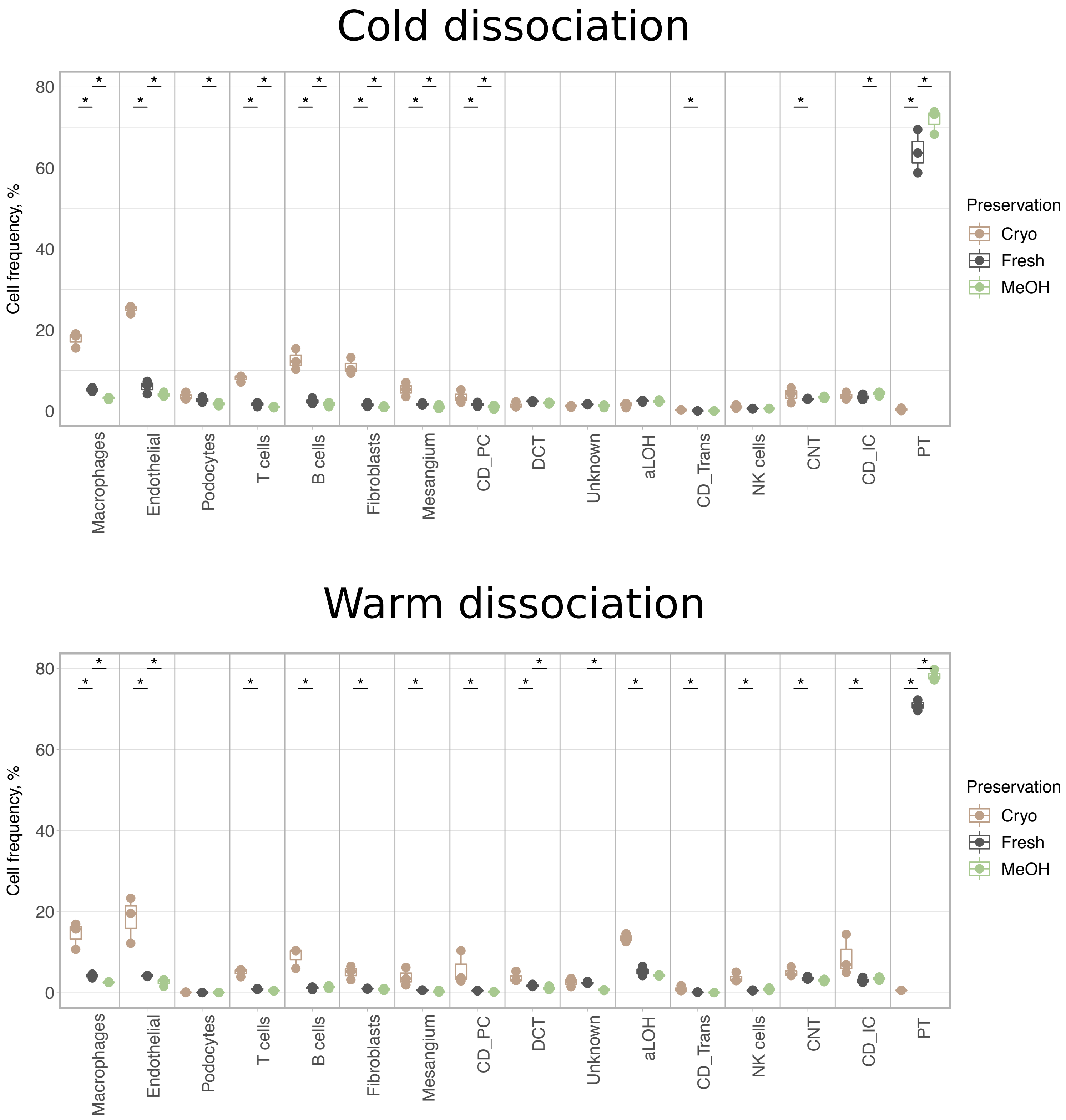


Cell type composition of fresh and preserved kidneys. Three biological replicates are shown per condition. Asterisks denote two-sided chi-square test p-value < 0.001.

aLOH: ascending loop of Henle; CD_IC: intercalated cells of collecting duct; CD_PC: principal cells of collecting duct; CD_Trans: transitional cells of collecting duct; CNT: connecting tubule; DCT: distal convoluted tubule; PT: proximal tubule.

# Fig. S6

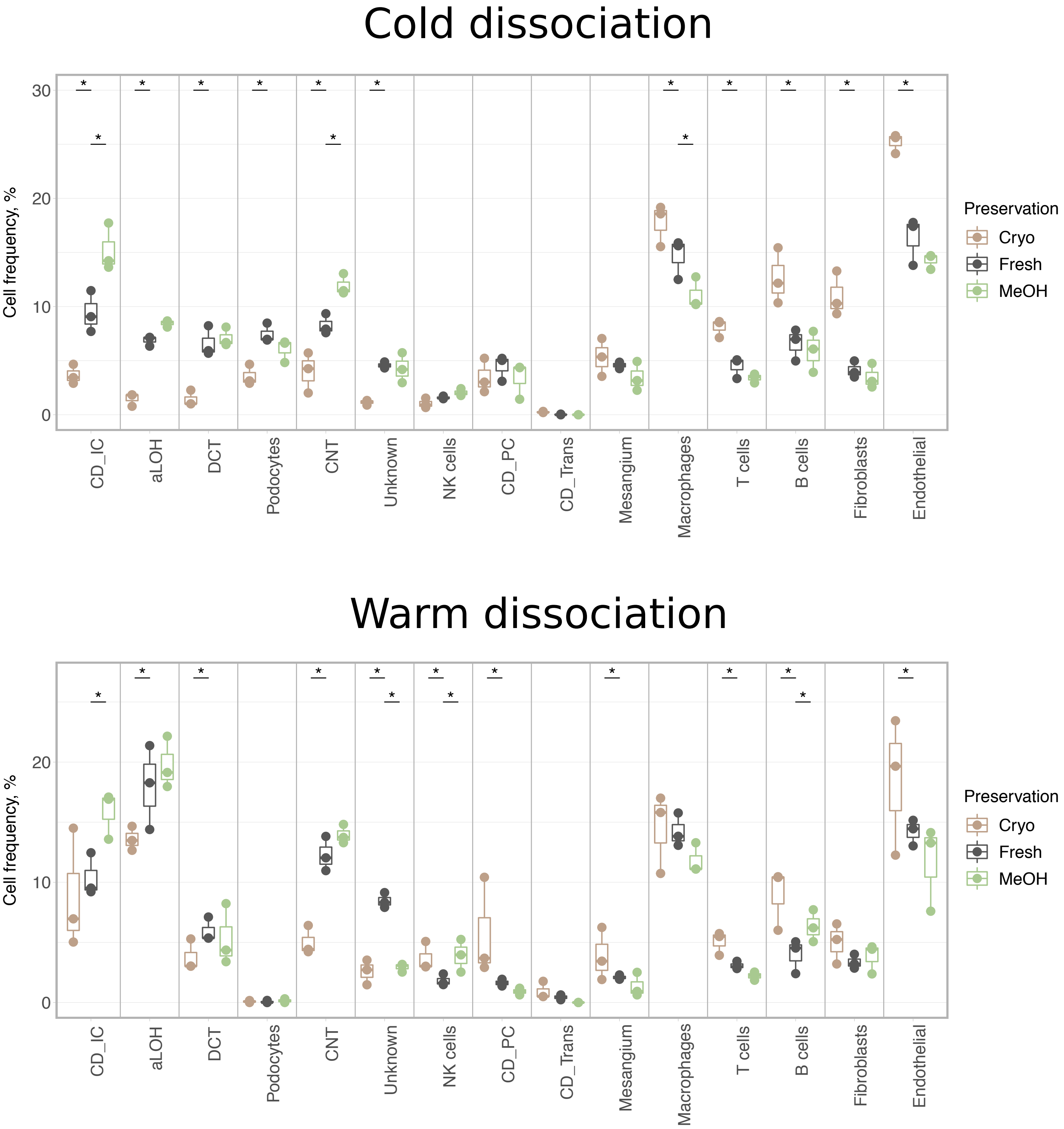


Cell type composition of non-proximal tubule cells in fresh and preserved kidneys. Three biological replicates are shown per condition. Asterisks denote two-sided chi-square test p-value < 0.001.
aLOH: ascending loop of Henle; CD_IC: intercalated cells of collecting duct; CD_PC: principal cells of collecting duct; CD_Trans: transitional cells of collecting duct; CNT: connecting tubule; DCT: distal convoluted tubule.

# Fig. S7

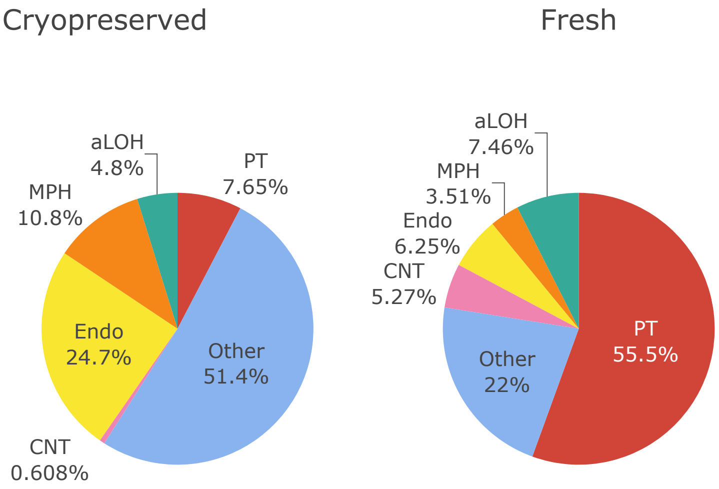


Cell type composition of fresh and cryopreserved cold-dissociated samples in the repeated experiment using Balb/c female mice, 10x v3 chemistry, 2 weeks storage, and 1200g spin.

aLOH: ascending loop of Henle; MPH: macrophages; CNT: connecting tubule; PT: proximal tubule.

# Fig. S8

A

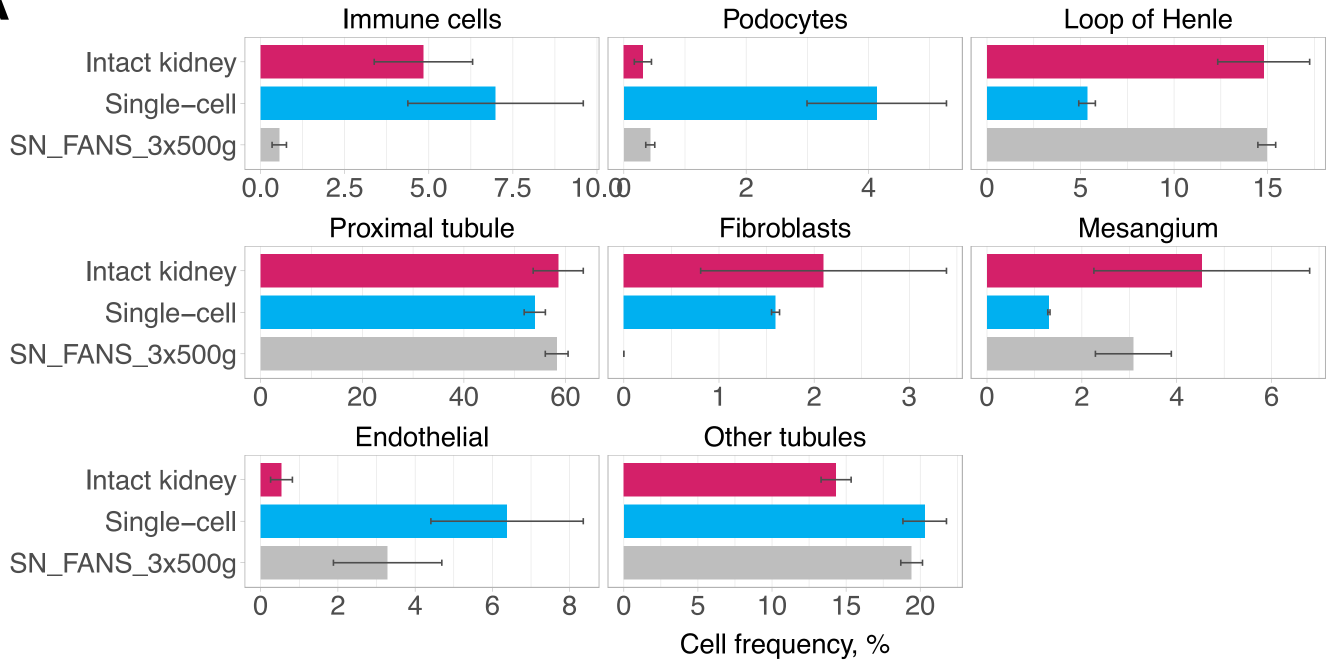


B

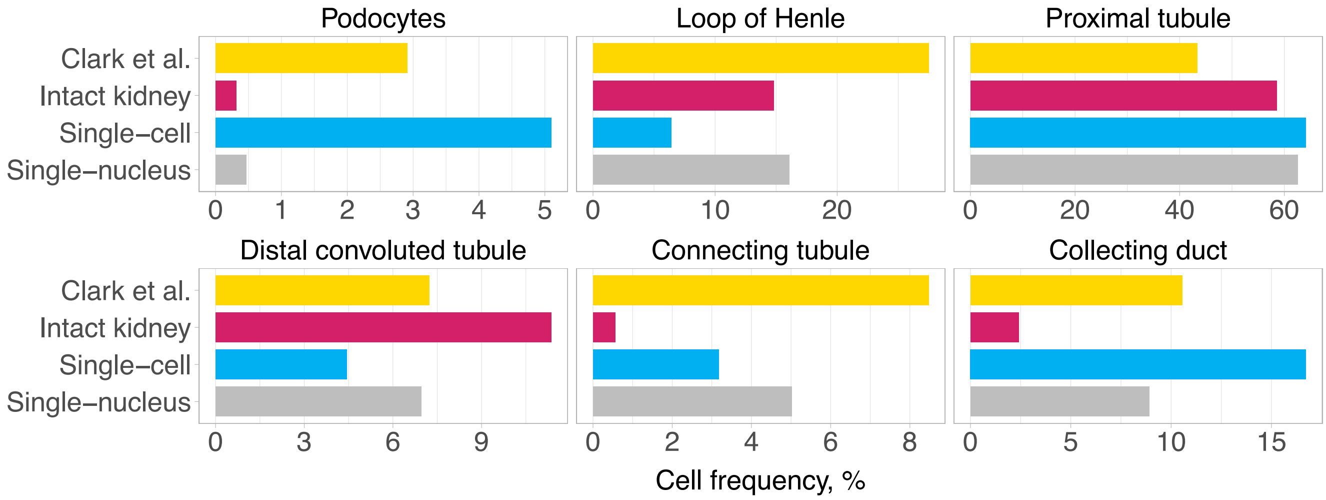


Comparison of single-cell and single-nucleus libraries in Balb/c male mice. **a**) Cell type composition for kidneys from Balb/c male mice. Average percentages for scRNA-seq libraries are shown in blue and for snRNA-seq libraries in grey. BSEQ-sc estimates are shown for bulk RNA-seq of intact kidneys. Error bars are standard error of mean. **b**) Abundance of renal epithelial cell types in Clark *et al.* study in comparison to our data from Balb/c male mice.

# Fig. S9

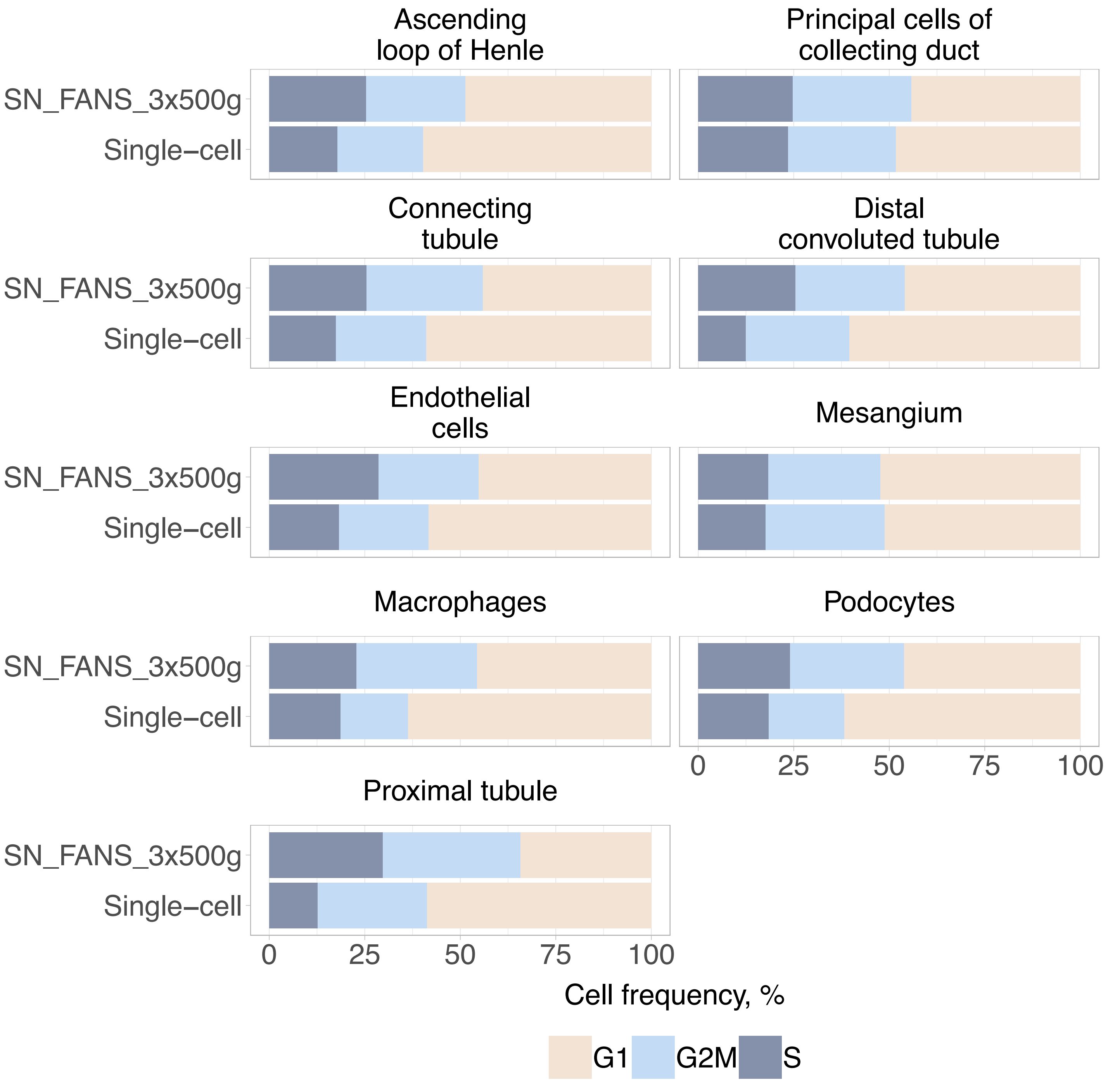


Cell cycle phases inferred in scRNA-seq and snRNA-seq libraries from Balb/c male mice.

# Fig. S10

1st gate for nuclei

2nd gate for singlets

FSC Par Area

3rd gate for DAPI+

Density plot

FANS gating strategy.

# Fig. S11

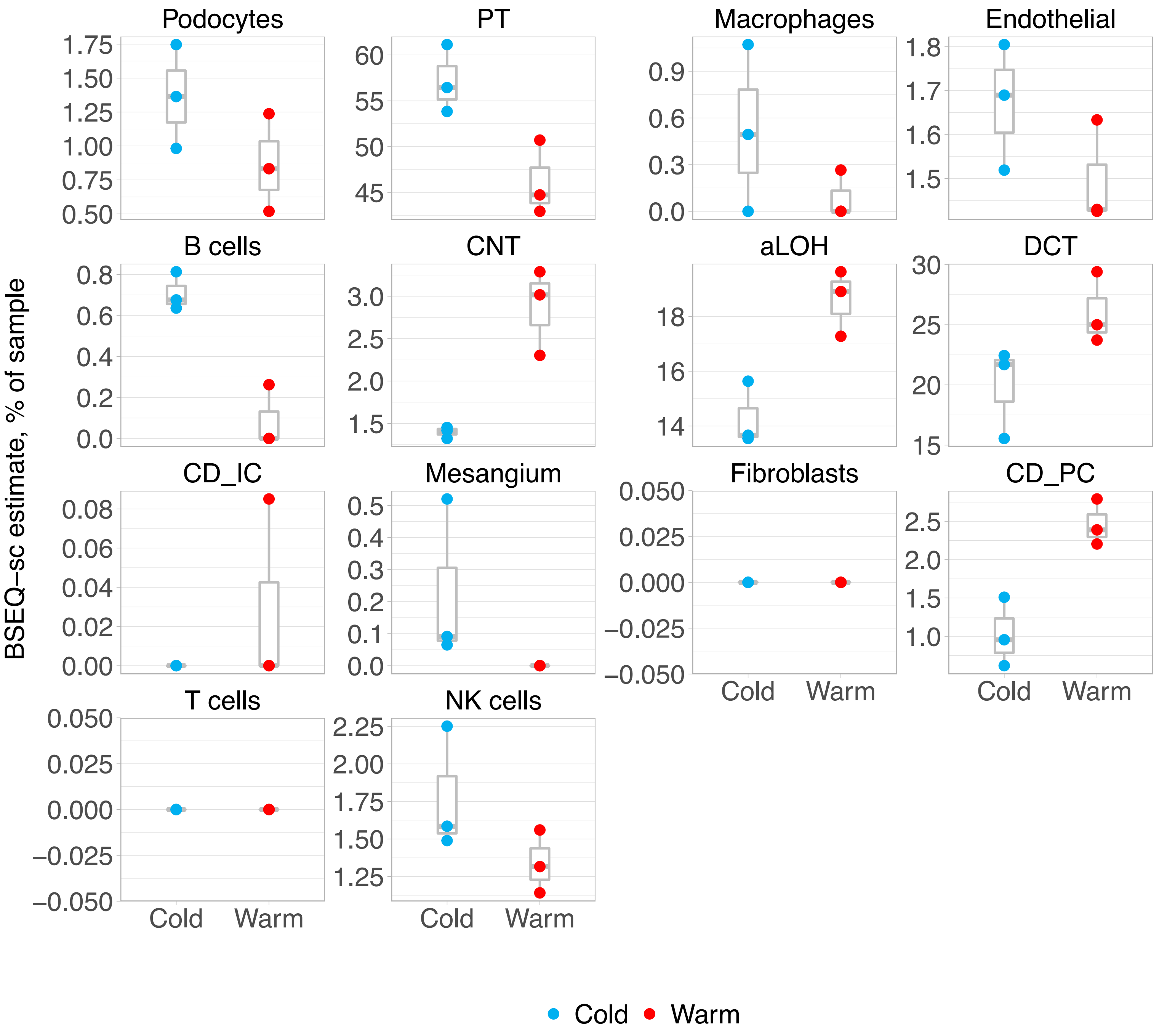


BSEQ-sc deconvolution of bulk RNA-seq profiles of cold- and warm-dissociated kidney single-cell suspensions. Three biological replicates are shown per condition.

aLOH: ascending loop of Henle; CD_IC: intercalated cells of collecting duct; CD_PC: principal cells of collecting duct; CNT: connecting tubule; DCT: distal convoluted tubule; PT: proximal tubule.

Fig. S12

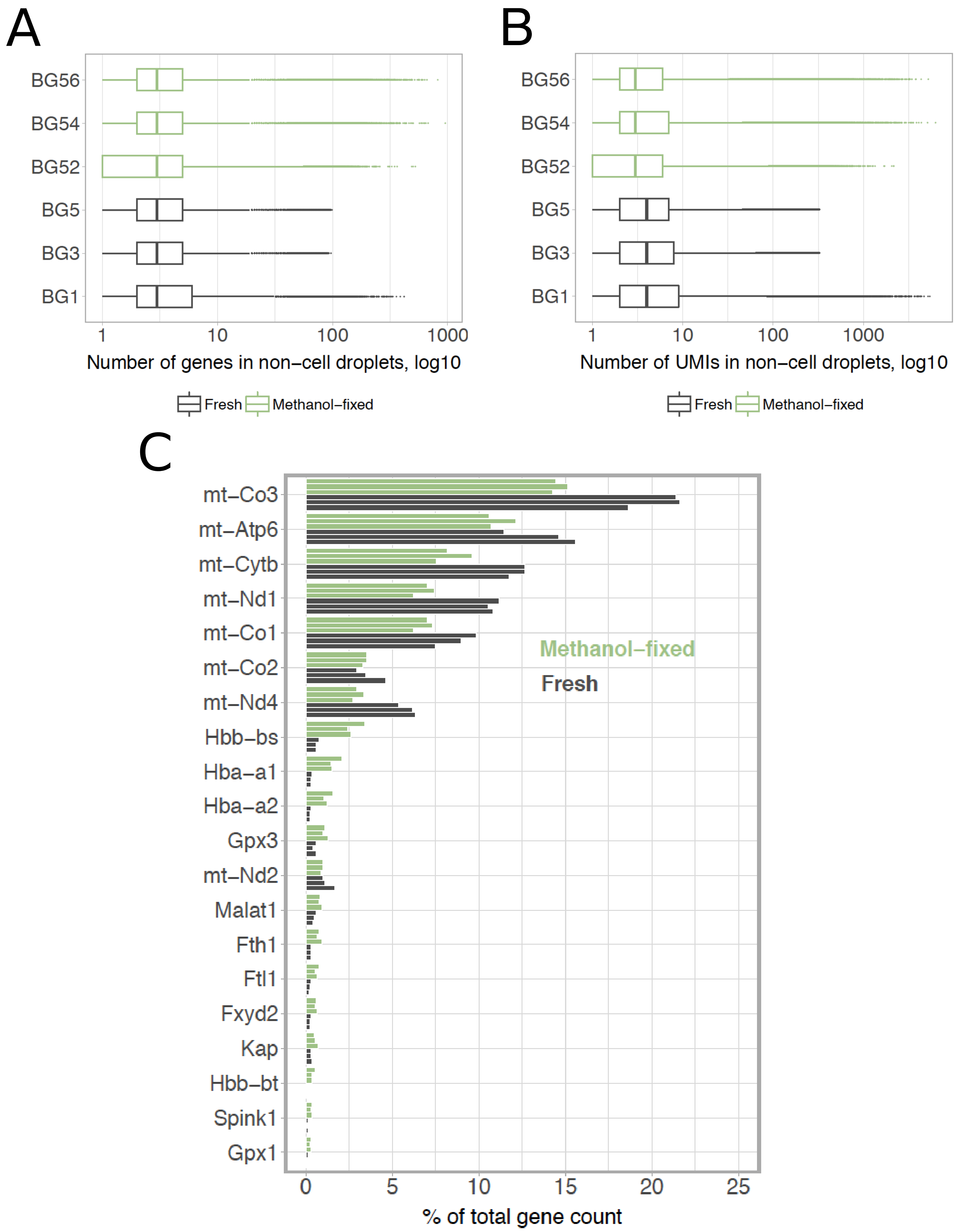


Comparison of ambient RNA contamination in methanol-fixed and freshly profiled aliquots of cold-dissociated samples.

# Fig. S13

A

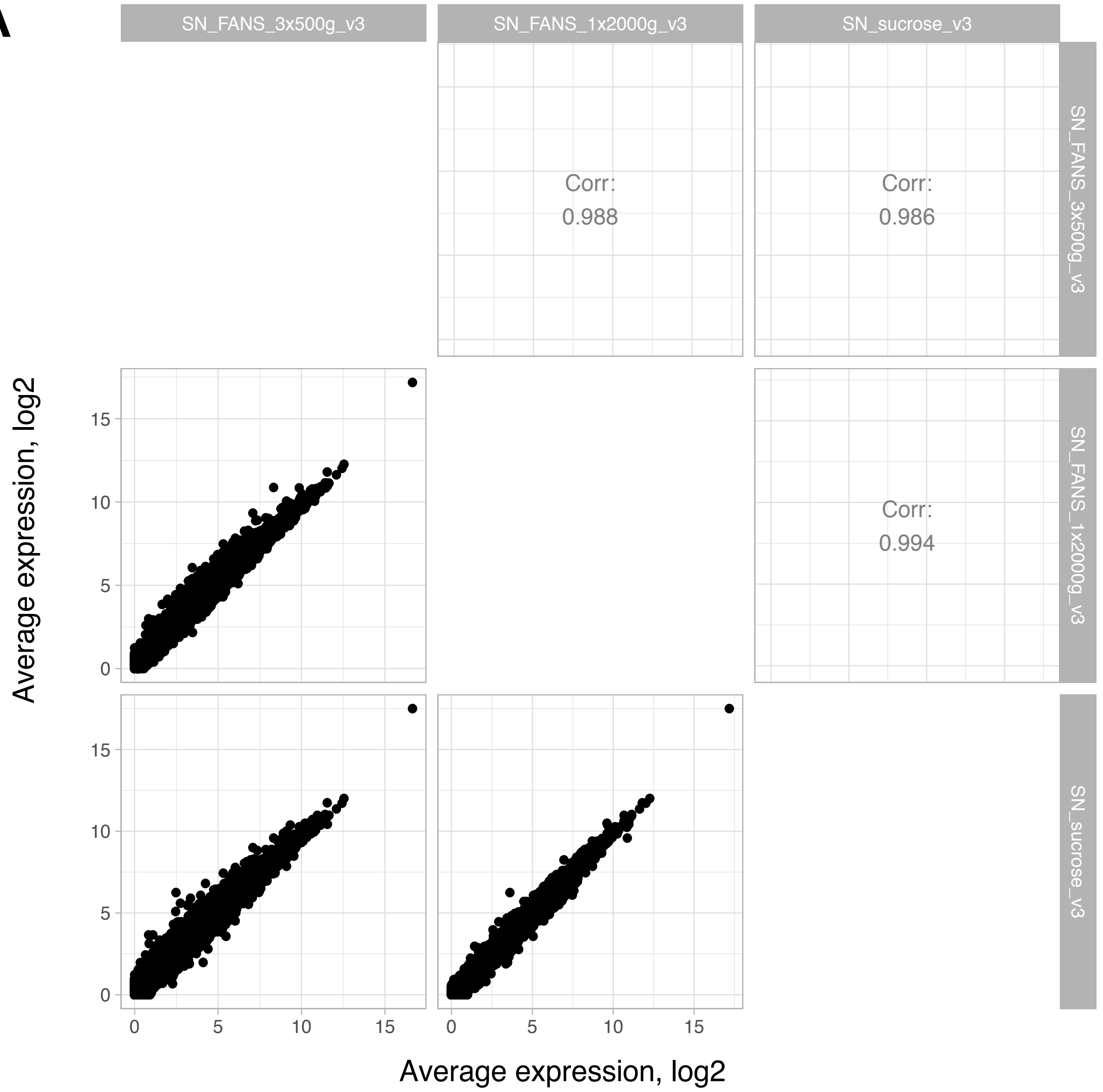


B

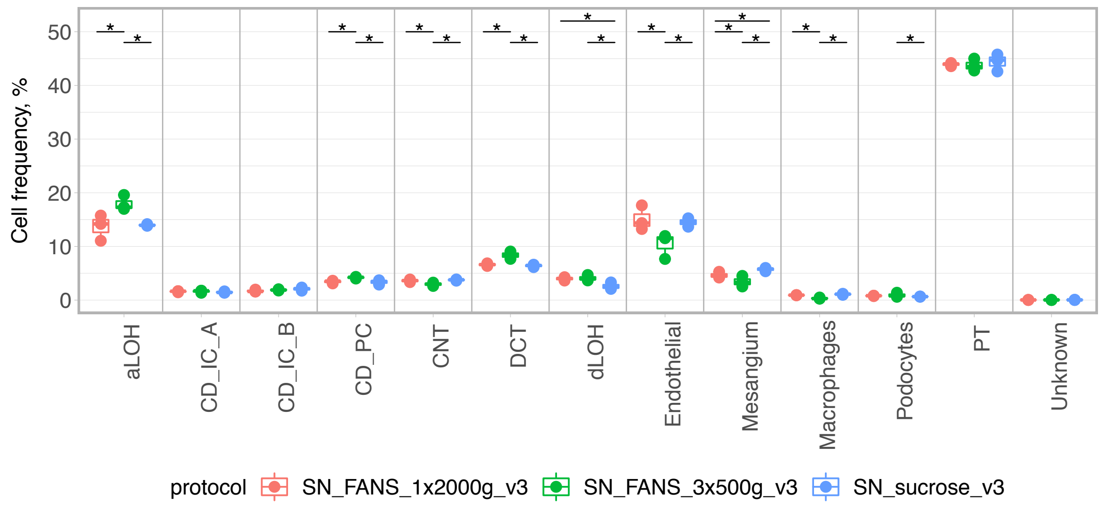


Comparison of nuclei isolation protocols. **a**) Aggregate gene expression and correlation between protocols. Raw counts were summed up for nuclei in each sample separately, then normalised to reads per million, averaged across biological replicates and log2-transformed with a pseudo count of 1 for plotting. Spearman correlation coefficients are shown. **b**) Cell type composition of the three nuclei isolation protocols. Three biological replicates are shown per protocol. Asterisks denote two-sided chi-square test p-value < 0.001. aLOH: ascending loop of Henle; CD_IC_A: type A intercalated cells of collecting duct; CD_IC_B: type B intercalated cells of collecting duct; CD_PC: principal cells of collecting duct; CNT: connecting tubule; DCT: distal convoluted tubule; dLOH: descending loop of Henle; PT: proximal tubule.

Fig. S14

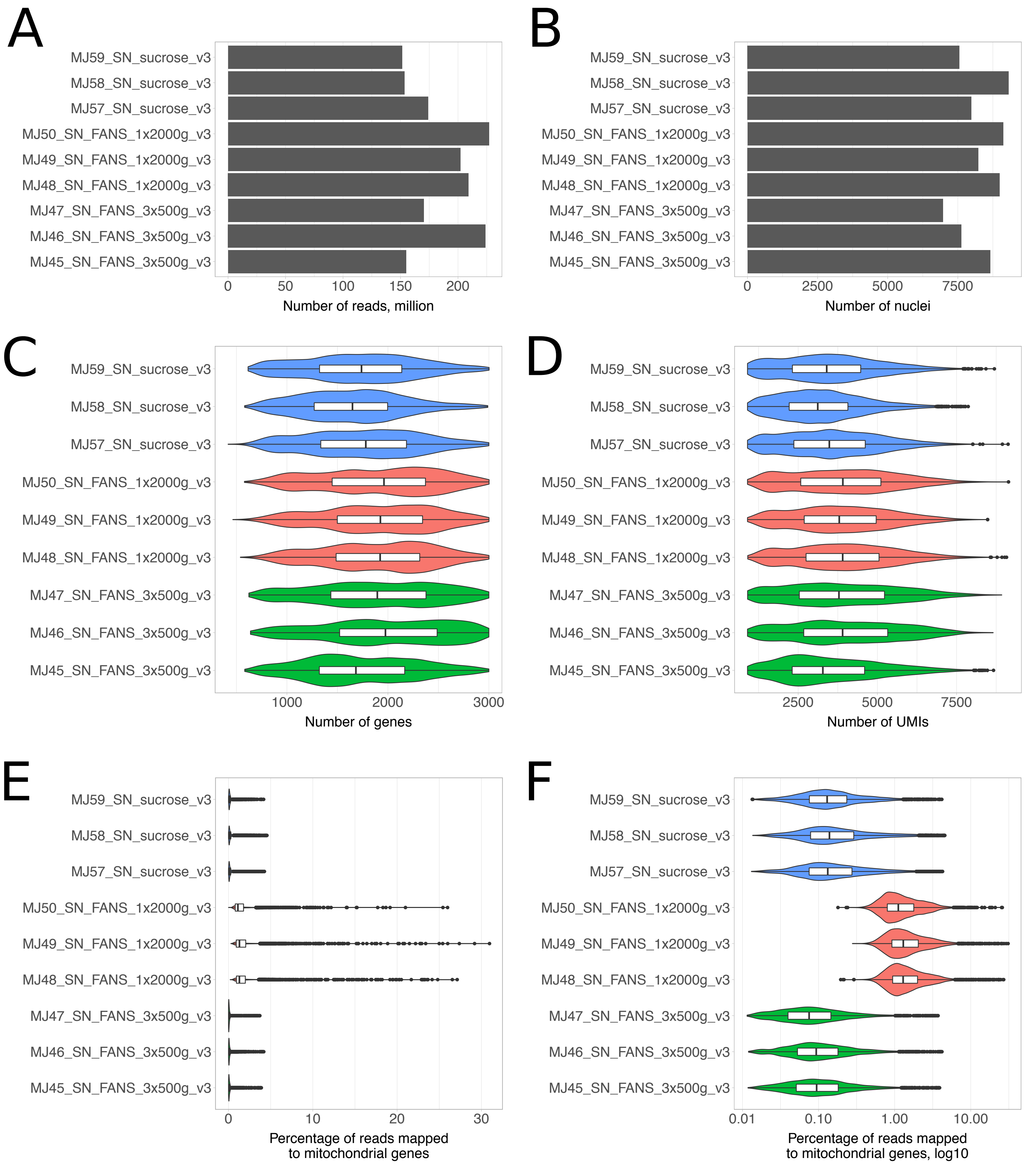


Comparison of nuclei isolation protocols. **a**) Total number of sequenced reads for each library. **b**) Number of nuclei passing all filtering steps as described in **Methods**. **c**) Distribution of the number of genes detected in nuclei. **d**) Distribution of the number of unique molecular identifiers detected in nuclei. **e**), **f**) Distribution of the percentage of reads mapped to mitochondrial genes.

# Fig. S15

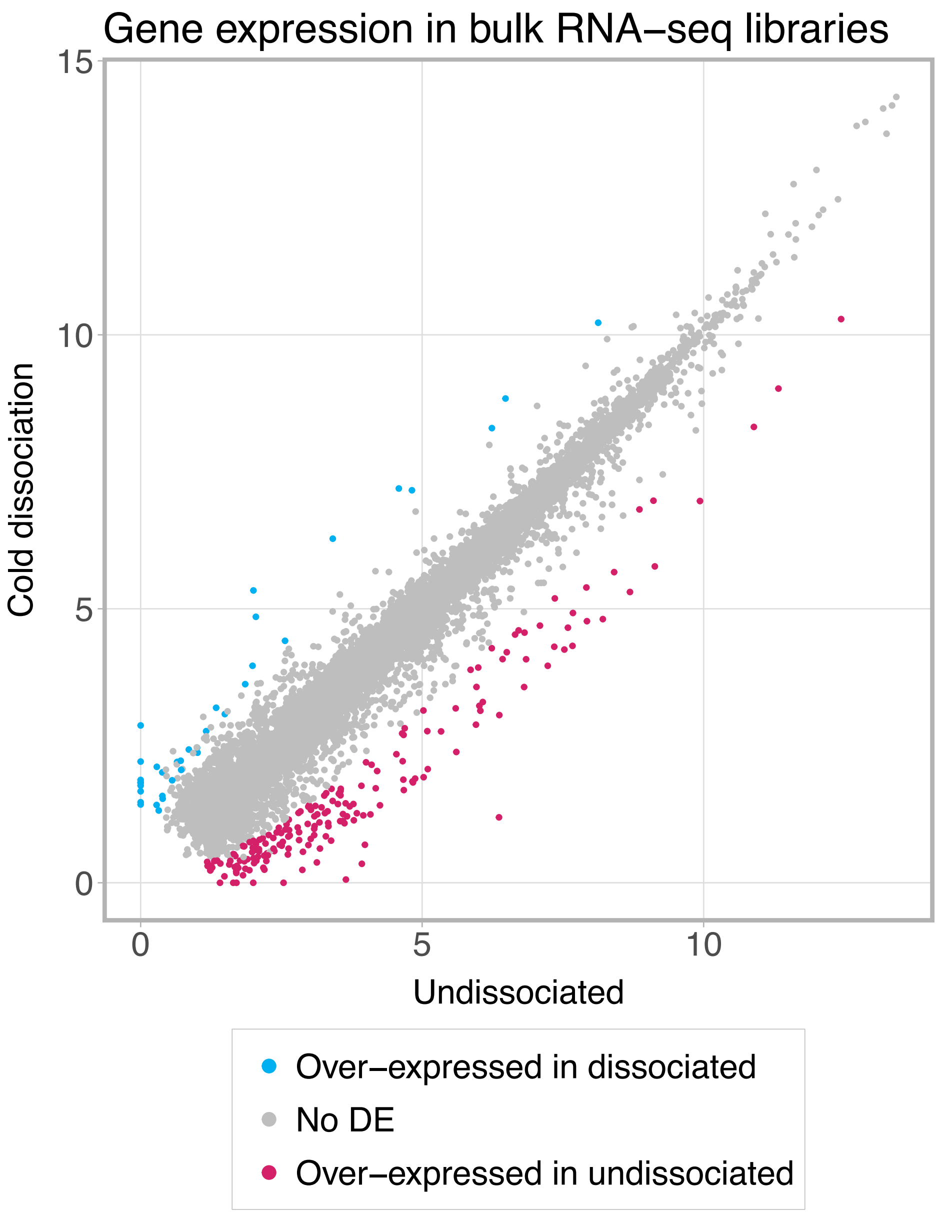


Comparison of bulk RNA-seq profiles of intact kidneys and cold-dissociated single-cell suspensions. GeTMM-normalised counts were averaged across three biological replicates and log2-transformed after adding a pseudo count of 1. DEGs identified with FDR < 0.05 and logFC threshold of 2 using edgeR exact test are indicated.

# Fig. S16

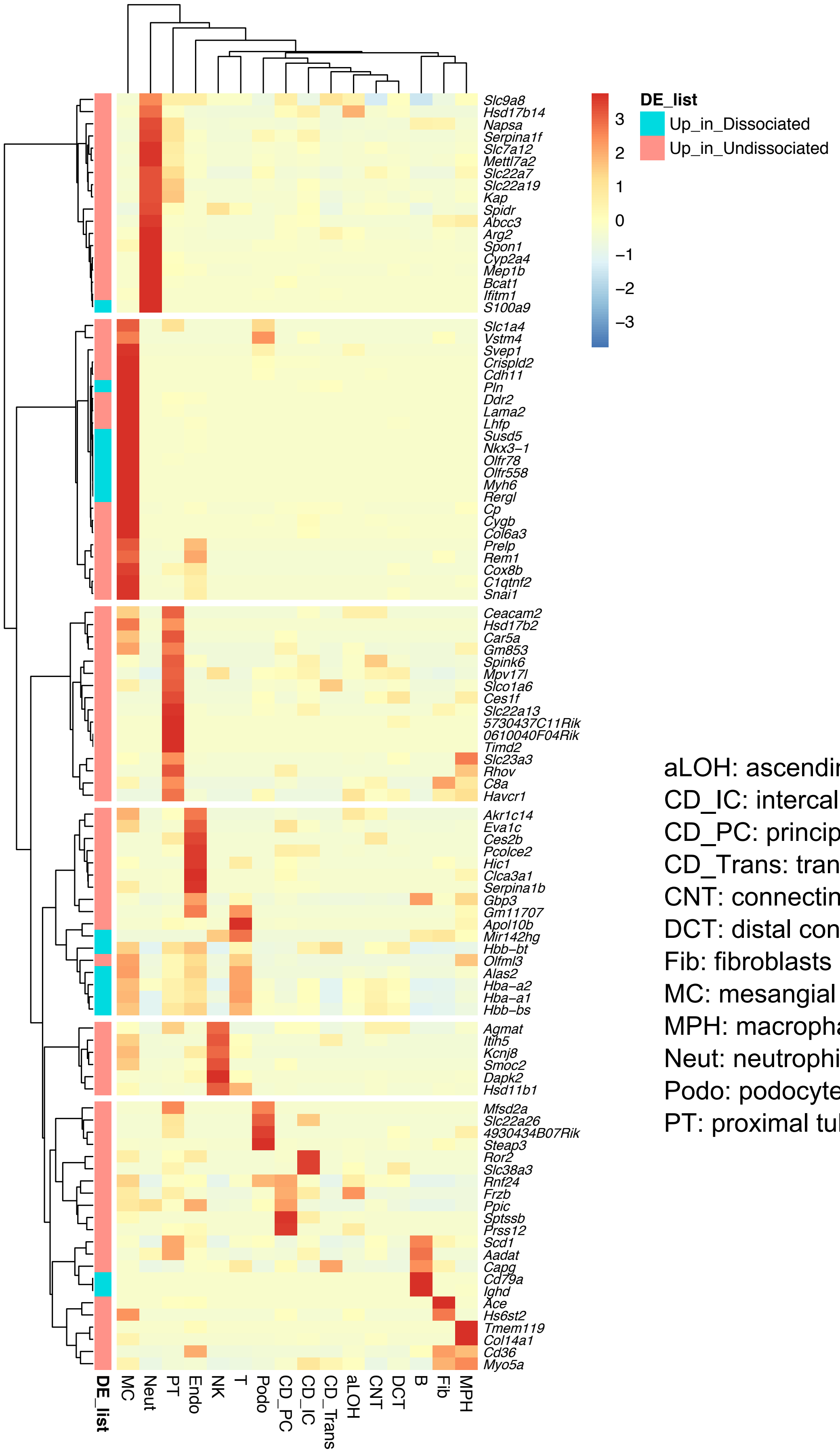


aLOH: ascending loop of Henle
CD_IC: intercalated cells of collecting duct
CD_PC: principal cells of collecting duct
CD_Trans: transitional cells
CNT: connecting tubule
DCT: distal convoluted tubule
Fib: fibroblasts
MC: mesangial cells
MPH: macrophages
Neut: neutrophils
Podo: podocytes
PT: proximal tubule

Expression of genes differentially expressed between bulk RNA-seq profiles of intact and dissociated kidneys in the matching single-cell dataset, Balb/c female mice. Normalised counts were averaged for each cell type, rows were scaled for plotting.
