# Supplementary material for: Systematic assessment of tissue dissociation and storage biases in single-cell and single-nucleus RNA-seq workflows
Source: Genome Biol. 2020 Jun 2;21:130. doi: 10.1186/s13059-020-02048-6 (PMC7265231; doi:10.1186/s13059-020-02048-6)
Supplement: Supplementary file 6 — Additional file 6. Supplementary Notes. [file 13059_2020_2048_MOESM6_ESM.pdf]

Supplementary Note 1. Microfluidics may alter cell composition.

To determine whether microfluidic partitioning could affect cell composition, we compared bulk RNA-seq and scRNA-seq data generated on the same dissociated kidney samples (**Fig. 1A, B**). We used BSEQ-sc [1] to predict the proportions of each cell type present in the samples before they were loaded on the microfluidics. Most notably, BSEQ-sc predicted cells of the ascending loop of Henle (aLOH) to be present at 18.6% and 14.3% on average for 3 biological replicates for warm and cold dissociation, respectively (**Additional file 2: Fig. S11**), while they were only present at 4.99% and 2.52% in the scRNA-seq libraries (warm- and cold-dissociated, respectively). Given that cells of aLOH are thought to be the second most populous cell type in kidney (estimated at 23.71% of kidney epithelial cells [2]), the analysis suggests the BSEQ-sc estimates are more likely to be correct and that aLOH cells may be lost in the microfluidic partitioning.

Podocytes were highly depleted in the warm dissociated samples (0.03% in warm vs 2.78% in cold); however, BSEQ-sc predicted podocytes to be at more similar abundance in the warm- and cold-dissociated samples (0.86% and 1.36%, respectively). In addition, known podocyte markers *Nphs1* and *Nphs2* were not significantly differentially expressed between bulk RNA-seq profiles of warm- and cold-dissociated kidneys (logFC = 0.397, FDR = 0.039 and logFC = 0.390, FDR = 0.032, respectively, edgeR exact test [3]). Together, this suggests that microfluidic partitioning likely contributes to the depletion of podocytes specifically in warm-dissociated kidneys.

Supplementary Note 2. Ambient RNA profile of methanol-fixed cold-dissociated kidneys.

We observed substantial contamination with highly abundant transcripts in methanol-fixed cell suspensions across all cell types (**Fig. 3D**). To investigate this phenomenon in more detail we selected all droplets which were discarded as empty ones by the EmptyDrops tool [4] in the cold-dissociated samples. **Additional file 2: Fig. S12A, B** shows boxplots comparing the numbers of genes and UMIs per empty droplet across fresh and methanol-fixed samples. Note that droplets with at least 1 gene detected are included. In methanol-fixed samples we find more outliers with over 100 genes (9715 droplets in total), whereas only one of the fresh samples has droplets with this many genes (1021 droplets in total). However, both figures indicate that the amount of contaminating ambient RNA does not seem to be dramatically higher in the methanol-fixed samples.

Next, we investigated what genes contribute to the ambient RNA profile. We again selected all ‘empty droplets’ and now summed up their counts for each sample to obtain aggregate ambient RNA counts. Then we calculated the percentage of counts attributed to each gene in each sample. **Additional file 2: Fig. S12C** shows the top 20 genes with the highest percentage in methanol-fixed samples. The figure indicates that mitochondrial genes compose a larger fraction of ambient RNA in fresh samples. In contrast, haemoglobin RNA and abundant tubular transcripts that are seen in **Fig. 3D** contribute more to the ambient RNA profile of methanol-fixed kidneys.

Hence, while the total amount of ambient RNA does not differ drastically between the fresh and methanol-fixed samples, we found differences in ambient RNA profile composition, which is ultimately seen in the downstream analysis as higher levels of haemoglobins and tubular genes in methanol-fixed cells.

Supplementary Note 3. Comparison of three nuclei isolation protocols.

Aggregate gene expression was highly correlated in the three nuclei isolation protocols (**Additional file 2: Fig. S13**) and each yielded similar numbers of nuclei with similar numbers of genes and UMIs detected per nucleus (**Additional file 2: Fig. S14)**. The major difference observed was a higher percentage of reads mapping to mitochondrial genes in the SN_FANS_1x2000g_v3 data (mean of 1.69%), vs 0.27% and 0.15% for the SN_sucrose and SN_FANS_3x500g_v3 data, respectively (**Additional file 2: Fig. S14**). **Additional files 11-13** list differentially expressed genes for three pair-wise comparisons between the protocols, calculated for each cell type separately using Wilcoxon test in Seurat [5] with thresholds of logFC = 0.5, minimum detection rate 0.5, FDR < 0.05. The differential expression analysis suggested that contamination of all cell populations with highly expressed kidney transcripts was the strongest in SN_FANS_1x2000g_v3 and the lowest in SN_FANS_3x500g_v3. In terms of cell type composition, SN_FANS_1x2000g_v3 and SN_sucrose were mostly similar, while SN_FANS_3x500g showed significant differences across several cell populations (**Additional file 2: Fig. S13**). Taking the low levels of contamination and the simpler protocol without need for specialist FANS equipment we recommend the SN_sucrose protocol.

Supplementary Note 4. Comparison of bulk RNA-seq of intact kidneys and cold-dissociated cell suspensions.

We compared bulk RNA-seq profiles of undissociated kidneys to bulk RNA-seq profiles of cold-dissociated single-cell suspensions derived from kidneys of Balb/c female mice. Raw counts were normalised to gene length and then to library sizes using weighted trimmed mean of M-values (TMM) method in edgeR [3], to derive gene length corrected trimmed mean of M-values (GeTMM) as described in [6] (see **Methods**). Note that single-cell suspensions were filtered through 70μm and 40μm cell strainers, hence, this comparison could potentially reveal poorly dissociated cell types.

Differential expression analysis (edgeR exact test [3] with FDR < 0.05 and logFC threshold of 2) identified 191 genes with higher expression in undissociated kidneys and 36 genes with higher expression in dissociated kidneys (**Additional file 2: Fig. S15**, **Additional file 15**). To get insight into the potential source of these genes, we investigated their expression levels in our single-cell dataset derived from the same batch of mice. Of a total of 227 differentially expressed genes, 26 genes were absent from the 10x transcriptome reference, hence, were not measured in single-cell experiments. Another 99 genes were not detected in the single-cell dataset, which could indicate either loss of certain cell types or low expression levels of these genes below the detection limit on a single-cell level. The latter is supported by the fact that genes which were detected in single-cell experiments had higher expression levels in undissociated bulk RNA-seq samples than genes not detected in single-cell experiments (mean expression of 200.9 versus 9.3 GeTMM-normalised counts, median 24.5 versus 4.1 GeTMM-normalised counts; two-sided Mann-Whitney test W = 7687, p-value < 2.2e-16). On the other hand, amongst the 99 genes not detected in the single-cell data, several are indicative of specific cell types. For example, the nervous tissue transcripts *Cck, Ak5* and *Gabra3* were more abundant in the intact kidney and may indicate RNA from nerve fibers which would not be expected to be seen in single-cell preparations.

Amongst the 102 differentially expressed genes which were detected in the single-cell dataset, 86 genes showed higher expression in undissociated kidneys and 16 – higher expression in dissociated suspensions. Amongst the 16 genes more abundant in the dissociated samples we identified haemoglobins (*Hbb-bs*, *Hba-a1*, *Hbb-bt*, *Hba-a2*) indicative of red blood cells. **Additional file 2: Fig. S16** shows a heatmap for average expression levels of the 102 genes in all cell types in the single-cell dataset. The heatmap reveals that several cell types, such as neutrophils, proximal tubules or endothelial cells, express genes which showed higher

expression in undissociated than dissociated kidneys. This might indicate that the corresponding cell types were incompletely dissociated. Surprisingly, mesangial cells express both genes which showed higher expression in undissociated kidneys and genes with higher expression in dissociated suspensions, which might point to mesangial cell subtypes that are unequally represented in intact vs dissociated bulk RNA-seq kidney profiles.

References

1. Baron M, Veres A, Wolock SL, Faust AL, Gaujoux R, Vetere A, et al. A single-cell transcriptomic map of the human and mouse pancreas reveals inter- and intra-cell population structure. Cell Syst. 2016;3:346-60.e4.
2. Clark JZ, Chen L, Chou CL, Jung HJ, Lee JW, Knepper MA. Representation and relative abundance of cell-type selective markers in whole-kidney RNA-Seq data. Kidney Int. 2019;95:787-96.
3. Robinson MD, McCarthy DJ, Smyth GK. edgeR: a Bioconductor package for differential expression analysis of digital gene expression data. Bioinformatics. 2010;26:139-40.
4. Lun ATL, Riesenfeld S, Andrews T, Dao TP, Gomes T, Marioni JC. EmptyDrops: distinguishing cells from empty droplets in droplet-based single-cell RNA sequencing data. Genome Biol. 2019;20:63.
5. Butler A, Hoffman P, Smibert P, Papalexi E, Satija R. Integrating single-cell transcriptomic data across different conditions, technologies, and species. Nat Biotechnol. 2018;36:411-20.
6. Smid M, Coebergh van den Braak RRJ, van de Werken HJG, van Riet J, van Galen A, de Weerd V, et al. Gene length corrected trimmed mean of M-values (GeTMM) processing of RNA-seq data performs similarly in intersample analyses while improving intrasample comparisons. BMC Bioinformatics. 2018;19:236.
