# Supplementary material for: Systematic assessment of tissue dissociation and storage biases in single-cell and single-nucleus RNA-seq workflows
Source: Genome Biol. 2020 Jun 2;21:130. doi: 10.1186/s13059-020-02048-6 (PMC7265231; doi:10.1186/s13059-020-02048-6)
Supplement: Supplementary file 22 — Additional file 22. Review history. [file 13059_2020_2048_MOESM22_ESM.docx]

Review History

**First round of review**

**Reviewer 1**

**Are you able to assess all statistics in the manuscript, including the appropriateness of statistical tests used?**

No, I do not feel adequately qualified to assess the statistics.

**Comments to author:**

This is an excellent study that carries out a systemic study of different methods of preparation of samples for single cell transcriptional analysis. ScRNA-seq is rapidly becoming a standard procedure in the lab, replacing bulk RNA-seq approaches that cannot determine which cell type expresses which transcript. It is the foundational technology for many large projects, such as the Human Cell Atlas consortium. It is therefore critically important to compare different sample preparation technologies and to use those that result in the fewest artifacts.

In this manuscript we see a comparison of cold dissociation, warm dissociation, three different single nuclear protocols, as well as cryopreservation and methanol fixation. The work is thorough, with very large numbers of cells analyzed. I found it very informative and think it would in general make a valuable contribution to the single cell field.

Nevertheless, there are some reservations. First, this paper is not the first to address these topics. Nuclear versus cell comparisons have been published before, as have cold versus warm comparisons, and other papers have described cryopreservation and methanol fixation. But this paper presents some novel findings in these areas. Cryopreservation is not perfect, as they find that some cells do not survive the freeze/thaw well. Their results on the cold versus warm dissociations are largely confirming previous publications, but with some wrinkles. And this is the first work to my knowledge that addresses all of these issues side by side.

The following comments need to be addressed:

Page five lines 2,3 note that there are higher levels of haemoglobin RNAs in the ice samples. One wonders what the significance of this observation is. Normally RBC contamination is minimized by use of RBC lysis buffer. I don’t see this in either of the protocols used, warm or cold.

Page 4 lines 21-29. How were the 17 stress genes selected? It is quite interesting that while the warm dissociation stress response observed by Adam et al is confirmed, there are some differences. In particular Adam et al observed significant gene expression differences in kidney cell types (e.g. PT) where this study found few. One could imagine that this is the result of the different ages of the mice used in the two studies (P1 versus adult) or the result of the different warm proteases used in the two studies. Maybe cells still in the midst of the kidney developmental program are more sensitive to stress. And/or some proteases might cause more stressful warm environments than others, thereby impacting more cell types.

Page 5 lines 16-18. It is suggested that there might be a preferential loss (warm versus cold) of some cell types during the microfluidic partitioning. The exact potential mechanism at work is not clear. A clogging issue would presumably then impact all cells. Is it thought that the cells are undergoing lysis during the microfluidics? This would then contribute to an ambient RNA (soup) issue. Was this seen? An interesting possibility is raised in the discussion, that these cells are not lysed. Could you see this by looking at the drops with a microscope?

Page 5 last paragraph. It is surprising that only 32% of cells were recovered after freeze/thaw, and that only 75% of these were viable. These numbers are low compared the experience of this reviewer. I do, however, agree with the results of the following paragraph, that not all cell types will survive freeze/thaw equally well, which is an important conclusion that is not widely appreciated. It is then stated that the cryopreservation experiments were repeated, due to the discord with previous published results. Were the low recovery/low viability again present in the repeat? This needs to be stated.

It is surprising that the cryopreserved samples show some stress response, singe the cryopreservation protocol takes place almost entirely on ice, or colder, where gene expression changes are not thought to take place.

I agree with the authors that the results of Figure 3 panel D look very much like a soup, ambient RNA issue. This should be examined more closely. On the knee curve how many genes called expressed are called for the barcodes at the right end of the graph that clearly did not have a cell? In our experience there is always soup, but it can vary dramatically, from 100 genes called expressed per empty drop, for an excellent cell prep, to well over a thousand for some nuclear preps.

Page 7 last paragraph. It is not surprising that the immune cells are showing higher representation in nuclear preps compared to cell. It is commonly observed that some immune cell types are very easily dissociated and are quite hardy. As a result, they are generally over represented in cell dissociation preps. On the other hand, the apparent loss of some immune cell types in certainly interesting and provides an argument for cells over nuclei.

The authors need to mention in the text the importance quality metric of genes called expressed/cell(nucleus) comparison of cell versus nuclei.

In general, it is important to note that cell compositions in single cell (or nuclear) data are never proportional to in vivo. Of course, one would like for as many as possible cell types to be represented in the single cell data, but for accurate proportionality determinations one needs to rely on other technologies, like in situ hybridizations or immuno stains.

**Reviewer 2**

**Are you able to assess all statistics in the manuscript, including the appropriateness of statistical tests used?**

Yes, and I have assessed the statistics in my report.

**Comments to author:**

The authors Denisenko, et al. present a detailed comparison of several tissue dissociation, preservation and cell/nucleus preparation techniques that were used to generate single cell transcriptome data (using the 10X Genomics platform) for mouse kidney. Good study design, there was a lot of appreciation given to reducing sample variation by using a single cell suspension (derived in different ways) for all of the proposed tests on both dissociation and preservation. As long as the samples were evenly sampled and mixed, one would expect any differences in cell type to be from the differences in handling. The study is well-designed and carefully executed.

Concerns:

Differences in dissociation may not be as drastic even if the dissociations come to different end results. Warm dissociation may be more complete, albeit with up-regulation of stress response genes. Perhaps the loss of weaker cell types may be ameliorated by removing cells that were already released into the dissociation/digestion media. They note that the cell types lost in cold dissociation were harder to dissociate cell types, which could indicate that to get them may warrant the loss of other cell types observed in the hot dissociation. One option would be to combine both hot and cold-acting digestions.

Current work supports the general findings of the single cell and Human Cell Atlas community and broadly reiterates that different tissue preservation, dissociation, and preparation methods tested each have pros and cons, making them all potentially usable under different scenarios.

When this is combined with the fact that this data may be hard to extrapolate to different tissues it can be difficult to make definitive conclusions about the representative fraction of each cell type present in the mouse kidney through transcriptomic data alone.

Given that one can expect heterogeneity between individual mice, sex-specific differences, etc. it is difficult to conclude anything from the current study other than that are many confounding factors. It is unclear as to how to interpret the results in context of mouse kidney (e.g., or all the different cell types % reported here, which one should we go with?) or translate the finding to human kidney, mouse strains, pathologies, etc.

How well do the current findings in tissue dissociation translate to other tissue types? How do they translate to diseased tissue? Can the results be generalizable to human tissue?

For example, there is growing evidence that there is loss of epithelial cells under cryopreservation, loss of immune cells in single nuclei protocols, are likely to hold true in other tissue/organs. Others (PT vs. aLOH cells) are likely not applicable outside the context of mouse kidney.

I am unsure if the stress response score suggested by the authors is a good metric in that as it looks like it just takes the highest stressed cells in a given sample as 1, which doesn't mean that those cells are necessarily much stressed. As in, the high stressed cells might just be lowly stressed cells being compared to basically unstressed cells. It may be used to compare cell types present in a given sample but cannot be used to compare stress responses in a given cell-type across samples prepared under a set of tissue dissociation/preservation conditions, which would be more meaningful. How does the stress score proposed here compare to other scoring?

In discussion, there is no clear conclusion from the different conditions tested- the reader would expect some guidance from the authors in interpreting the multiple datasets generated under different conditions. Each seems to have pros and cons depending on which cell type(s) one might be interested in. Some discussion about the uses and pitfalls of both might be appropriate.

So there are differences: how to correct for them? There are different computational methods (Canonical Correlation Analysis, Mutual Nearest Neighbor, k-nearest-neighbor batch-effect test, etc.) to correct for batch effects and other technical artifacts that are not tested or compared here. Can they be used to combine the different datasets generated from the different techniques tested) be to generate a consensus data-set? This may fix the shortcomings of the different preservation/dissociation/preparation methods? Also, for the cells that are preferentially adversely affected by the preps, can the stress response genes or modules be regressed out?

Computational techniques to address such issues are not used on this well-designed experiment. Other than the use of scMatch, computational analyses were insufficient. Much more can be learned from such a carefully planned, exhaustive set of experiments. Why not?

A lot of the cell numbers we see comparing techniques don't come with comments on significance or values to show significance. In the absence of conclusive numbers for cell-type representation or expression, alternative techniques like FACS sorting on dissociated cells, or IHC on tissues may be used as orthogonal approaches to establish cell-type % and their gene expression.

Of the stress response genes identified in the study, are there any that are specific to the kidney or are they generally applicable to other tissue types?

It is unclear if the cold dissociation is indeed less stressful or if the lower temperature is suppressing the transcriptional machinery in general. In parallel, it is unclear if the 37oC incubation per se in the warm dissociation caused stress response or it is the presence of the tissue/cells outside their native/ physiological environment that lead to stress response. 37C is after all normal physiological temperature.

Minor concerns:

- Unclear as to what the authors mean when they propose that the aLOH cells are being lost in the microfluidics (p5, l16). Please clarify.

- How intact were the cells after running the tissue through the octoMacs? The octoMacs destroys tissue, so how are they ensuring that the cells are intact? Perhaps the octoMacs is shredding up more delicate cell types that they didn't catch?

- What was the viability of the cells?

- How did the authors identify the stopping point for the different enzymatic digestions? Are there any guidelines for visually distinguishing cells from debris during dissociation?

- Did they filter out any undissociated clumps during their cell/nuclei prep steps?

- Why not any other FBS/DMSO mixes? Is the epithelial cell loss consistent across all freezing media?

- Optical microscopy images of the final cell/nucleus suspensions obtained from each of the techniques tested would be very useful to visually assess the diversity in cell types, presence of debris, etc.

- How did the authors identify the end point of tissue digestion? Were different incubation times tested?

- Reasoning behind "higher fraction of recovered PT cells (7.65% vs. 0.57%) in the repeated experiment" (pg 12, l2) is hand-wavy. Perhaps the effect of worse chemistry in v2 (that would translate to lower capture before capture) can be simulated with down-sampled data and compared?

- Ambient RNA contamination can be checked with 'EmptyDrops' analysis (DropletUtils package).

**Authors Response**

**Point-by-point responses to the reviewers’ comments:**

*We thank the reviewers for the great suggestions and comments. We have endeavoured to address all of the reviewers concerns and believe the manuscript is greatly improved from their suggestions.*

*One note. In response to reviewer 2 minor point Q2-17 we had planned to carry out additional cryostorage experiments testing alternative cryostorage media but due to the ongoing COVID-19 crisis and the shut-down of our lab we have not been able to carry these out.*

Reviewer #1

This is an excellent study that carries out a systemic study of different methods of preparation of samples for single cell transcriptional analysis. ScRNA-seq is rapidly becoming a standard procedure in the lab, replacing bulk RNA-seq approaches that cannot determine which cell type expresses which transcript. It is the foundational technology for many large projects, such as the Human Cell Atlas consortium. It is therefore critically important to compare different sample preparation technologies and to use those that result in the fewest artifacts.

In this manuscript we see a comparison of cold dissociation, warm dissociation, three different single nuclear protocols, as well as cryopreservation and methanol fixation. The work is thorough, with very large numbers of cells analyzed. I found it very informative and think it would in general make a valuable contribution to the single cell field.

Nevertheless, there are some reservations.

First, this paper is not the first to address these topics. Nuclear versus cell comparisons have been published before, as have cold versus warm comparisons, and other papers have described cryopreservation and methanol fixation. But this paper presents some novel findings in these areas. Cryopreservation is not perfect, as they find that some cells do not survive the freeze/thaw well. Their results on the cold versus warm dissociations are largely confirming previous publications, but with some wrinkles. And this is the first work to my knowledge that addresses all of these issues side by side.

The following comments need to be addressed:

Q 1-1. Page five lines 2,3 note that there are higher levels of haemoglobin RNAs in the ice samples. One wonders what the significance of this observation is. Normally RBC contamination is minimized by use of RBC lysis buffer. I don’t see this in either of the protocols used, warm or cold.

*A 1-1. We thank the reviewer for bringing this to our attention. We have not used any of the red blood cell depletion approaches before sample loading, as we aimed to reduce adding extra steps to the protocols. Notably, RBC depletion can not be applied to the kidneys frozen for snRNA-seq.*

*We believe that the observed higher contamination in cold-dissociated samples might be the result of partial degradation of haemoglobin mRNA in warm dissociation conditions.*

*We updated the manuscript to reflect this by adding the following to the Discussion:*

*“Cold-dissociated samples showed higher contamination with haemoglobin transcripts than warm-dissociated ones. We note that red blood cell depletion methods were not applied in any of the experiments performed here and the observation likely stems from a higher rate of haemoglobin transcript degradation in warm tissue dissociation conditions.”*

Q 1-2. Page 4 lines 21-29. How were the 17 stress genes selected? It is quite interesting that while the warm dissociation stress response observed by Adam et al is confirmed, there are some differences. In particular Adam et al observed significant gene expression differences in kidney cell types (e.g. PT) where this study found few. One could imagine that this is the result of the different ages of the mice used in the two studies (P1 versus adult) or the result of the different warm proteases used in the two studies. Maybe cells still in the midst of the kidney developmental program are more sensitive to stress. And/or some proteases might cause more stressful warm environments than others, thereby impacting more cell types.

*A 1-2. To select the 17 genes, we looked at all genes induced in the warm-dissociated samples and from that list we manually selected genes which have been reported in the literature as stress-response-related genes. This method is data-driven and the only purpose here was to quantitatively compare the observed stress response between different cell types in this particular dataset. We updated the Methods section to make this clearer:*

“To select genes for the stress response score, we looked at all genes induced in the warm-dissociated samples and from that list we manually selected genes which have been reported in the literature as stress-response-related genes.”

In relation to the differences with the genes identified by Adam et al. (Figure 3. https://dev.biologists.org/content/144/19/3625.long). The authors examined heat response at 3 different time points (15m, 30m, 1h) in P1 mice whereas we only used a single time point from adult mice. There are also other differences, e.g. Adam et al. used various proteases (collagenase, ProNase E, DNAse, CaCl2 in DPBS) and manual trituration, whereas we used a commonly used commercial kit (Miltenyi Multi Tissue Dissociation Kit 2) with a proprietary enzyme mix. Notably however 10 of our 17 stress response signature genes are also seen in the Adam et al. analysis (their Fig 3).

Q 1-3. Page 5 lines 16-18. It is suggested that there might be a preferential loss (warm versus cold) of some cell types during the microfluidic partitioning. The exact potential mechanism at work is not clear. A clogging issue would presumably then impact all cells. Is it thought that the cells are undergoing lysis during the microfluidics? This would then contribute to an ambient RNA (soup) issue. Was this seen? An interesting possibility is raised in the discussion, that these cells are not lysed. Could you see this by looking at the drops with a microscope?

*A 1-3. We thank the reviewer for pointing this out. According to our analysis in Supplementary Note 1, bulk RNA-seq data suggest that proportions of aLOH cells in cell suspensions are higher than those observed in single-cell data, at 18.6% (warm, bulk) and 14.3% (cold, bulk) versus 4.99% (warm, single-cell) and 2.52% (cold, single-cell). However, the percentage is still higher for warm-dissociated kidneys than cold-dissociated kidneys in both single-cell and bulk data.*

*We have removed the corresponding sentence from the manuscript and replaced it with a separate paragraph:*

*“Finally, to determine whether microfluidic partitioning could affect cell composition, we compared bulk RNA-seq and scRNA-seq data generated on the same dissociated kidney samples (Supplementary Note 1). Our results suggest that proportions of aLOH cells in cell suspensions were higher before they were loaded on the Chromium Controller and that they are somehow underrepresented. As to possible mechanisms for this, it may be that they are more resistant to lysis in the device or that there is differential sampling of these cells due to cell size or shape as they enter the microfluidic device.”*

*However, in podocytes we did observe what might be a preferential loss. Fractions of podocytes were at 0.03% in warm single-cell and at 2.78% in cold single-cell, whereas in bulk RNA-seq we estimated 0.86% and 1.36%, respectively. In addition, known podocyte markers Nphs1 and Nphs2 were not significantly differentially expressed between bulk RNA-seq profiles of warm- and cold-dissociated kidneys (logFC = 0.397, FDR = 0.039 and logFC = 0.390, FDR = 0.032, respectively, edgeR exact test, Supplementary Note 1).*

In this particular case, given that podocytes are apparently easily damaged by warm dissociation, one could hypothesize that the warm-dissociated podocytes undergoing lysis are captured by bulk RNA-seq but not by 10x. To further investigate this possibility, we analysed the total mRNA pool derived from the 10x experiments. Specifically, we took the raw single-cell count data for 3 cold and 3 warm samples (i.e. including ‘empty’ droplets containing ambient RNA) and summed up the counts to obtain pseudo-bulk count profiles for each sample. We then compared cold- and warm-dissociated samples, however, podocyte markers Nphs1 and Nphs2 showed significantly lower expression in warm-dissociated samples.

Q 1-4. Page 5 last paragraph. It is surprising that only 32% of cells were recovered after freeze/thaw, and that only 75% of these were viable. These numbers are low compared the experience of this reviewer. I do, however, agree with the results of the following paragraph, that not all cell types will survive freeze/thaw equally well, which is an important conclusion that is not widely appreciated. It is then stated that the cryopreservation experiments were repeated, due to the discord with previous published results. Were the low recovery/low viability again present in the repeat? This needs to be stated.

*A 1-4. We thank the reviewer for this suggestion and now we have added the following to the Results section:*

*“Notably, only ~33% and ~32% of cells were recovered after cryostorage in the first and the repeated experiment, respectively; the average viability estimated by the Countess was 86% and 75%, respectively.”*

Q 1-5. It is surprising that the cryopreserved samples show some stress response, since the cryopreservation protocol takes place almost entirely on ice, or colder, where gene expression changes are not thought to take place.

*A 1-5. We believe transcription of stress-response-related genes might occur during cell thawing. Possibly, when thawing in the 37˚C water bath, cells on the outside that thaw first may be ‘heated’ more than the ones still frozen in the middle. We do try to mitigate this by swirling them around gently.*

Q 1-6. I agree with the authors that the results of Figure 3 panel D look very much like a soup, ambient RNA issue. This should be examined more closely. On the knee curve how many genes called expressed are called for the barcodes at the right end of the graph that clearly did not have a cell? In our experience there is always soup, but it can vary dramatically, from 100 genes called expressed per empty drop, for an excellent cell prep, to well over a thousand for some nuclear preps.

*A 1-6. Thank you for the suggestion. In the present work we have used the EmptyDrops package to distinguish real cells from empty droplets. To address the reviewer’s comment, we selected all droplets which were discarded as empty ones by this tool and investigated genes detected in those droplets.*

*Below are boxplots comparing numbers of genes per empty droplet across fresh and methanol-fixed samples. Note that only droplets with at least 1 gene detected are included. Freshly profiled samples are shown in grey (BG1, BG3, BG5), methanol-fixed samples are in green (BG52, BG54, BG56).*

*In methanol-fixed samples we find more outliers with over 100 genes (9715 droplets in total), whereas only one of the fresh samples has droplets with this many genes (1021 droplets).*

*Another figure below shows results of the same type of analysis for the total number of UMIs rather than genes detected per empty droplet:*

*Both figures indicate that the amount of contaminating RNA (‘soup’) does not seem to be dramatically higher in methanol-fixed samples, which is an unexpected observation given the findings from our downstream analysis. However, we next investigated what genes contribute to the ambient RNA profile and this revealed the differences between the two conditions.*

*We selected all ‘empty droplets’ and summed up their counts for each sample to obtain aggregate ambient RNA counts. Then we calculated the percentage of counts attributed to each gene in each sample. The figure below shows the top 20 genes with the highest percentage in methanol-fixed samples. The figure indicates that mitochondrial genes compose a larger fraction of ambient RNA in fresh samples. In contrast, haemoglobin RNAs and abundant transcripts from tubule cells that are seen in Figure 3D contribute more to the ambient RNA profile of methanol-fixed kidneys.*

*Hence, while the total amount of ambient RNA does not differ drastically between the fresh and methanol-fixed samples, we observe differences in ambient RNA profile composition, which is ultimately seen in the downstream analysis as higher levels of haemoglobins and tubular genes in methanol-fixed cells.*

*We have now added this analysis to the manuscript as Supplementary Note 2.*

Q 1-7. Page 7 last paragraph. It is not surprising that the immune cells are showing higher representation in nuclear preps compared to cell. It is commonly observed that some immune cell types are very easily dissociated and are quite hardy. As a result, they are generally over represented in cell dissociation preps. On the other hand, the apparent loss of some immune cell types is certainly interesting and provides an argument for cells over nuclei.

*A 1-7. We agree with the reviewer, and there is a sentence in the Discussion section reflecting this: “As Wu et al. have suggested, although these differences might indicate under-estimation of immune cells by snRNA-seq, another plausible explanation is that immune cell content is inflated in single-cell experiments as other cell types may be underrepresented due to incomplete dissociation”.*

*With regards to the lost cell types, one possibility is that they are indeed lost/damaged by snRNA-seq protocols. On the other hand, these cell types are very rare in the single-cell data, hence, if they are over-represented in the single-cell data, their numbers in single-nuclei preparations could drop down to virtually undetectable levels of just a handful of cells.*

Q 1-8. The authors need to mention in the text the importance quality metric of genes called expressed/cell(nucleus) comparison of cell versus nuclei.

*A 1-8. Thank you for the suggestion. We added the following sentence to the Results section:*

*“Single-nuclei sequencing detected more genes per nuclei than single-cell sequencing per cell, with the median numbers of 1819 and 981 genes, respectively (genes detected in at least 10 cells/nuclei were retained in each sample).”*

Q 1-9. In general, it is important to note that cell compositions in single cell (or nuclear) data are never proportional to in vivo. Of course, one would like for as many as possible cell types to be represented in the single cell data, but for accurate proportionality determinations one needs to rely on other technologies, like in situ hybridizations or immuno stains.

*A 1-9. The reviewer raises an important point. We added the following to the Discussion section:*

*“It is important to remember that cell type composition of single-cell or single-nucleus data differs from that of the original tissue and complementary approaches may be considered to improve the accuracy of the estimates. We note that even ISH/IHC/spatial transcriptomic methods, unless carried out on sufficient serial sections to completely survey the tissue of interest, will only give cellular proportions for the sections examined and will not give a whole (3D) organ estimate. Similarly, flow cytometry would help as an orthogonal approach to quantify cellular proportions within single cell suspensions but still requires the organ to be dissociated, which as we have shown here has biases. Although in vitro cell mixtures, or cell spiking experiments could be used to introduce different cell types at known ratios, we have not employed them in these experiments as they do not represent a true scenario of dissociation from a solid tissue”*

Reviewer #2

The authors Denisenko, et al. present a detailed comparison of several tissue dissociation, preservation and cell/nucleus preparation techniques that were used to generate single cell transcriptome data (using the 10X Genomics platform) for mouse kidney. Good study design, there was a lot of appreciation given to reducing sample variation by using a single cell suspension (derived in different ways) for all of the proposed tests on both dissociation and preservation. As long as the samples were evenly sampled and mixed, one would expect any differences in cell type to be from the differences in handling. The study is well-designed and carefully executed.

Concerns:

Q 2-1. Differences in dissociation may not be as drastic even if the dissociations come to different end results. Warm dissociation may be more complete, albeit with up-regulation of stress response genes. Perhaps the loss of weaker cell types may be ameliorated by removing cells that were already released into the dissociation/digestion media. They note that the cell types lost in cold dissociation were harder to dissociate cell types, which could indicate that to get them may warrant the loss of other cell types observed in the hot dissociation. One option would be to combine both hot and cold-acting digestions.

*A 2-1. We thank the reviewer for the comments. The aim of the work was to highlight that each dissociation strategy has its own inherent set of biases. Researchers starting on a new single cell project need to weigh these up for their own samples of interest. The cold dissociations do reduce the stress response and thus give a broader window of time for sequential dissociations (take cells that come off first and then continue to incubate). Sequential dissociations at 37˚C have issues as different cells will have had longer periods of time to induce a stress response (as reported by Adam et al.), and computationally figuring out which cells were exposed longer will be difficult.*

*The single nuclei, cryostorage, and methanol fixation options are all useful where storage of samples is needed (for example collecting patient tumour samples and waiting on pathology results), but as we have shown here they all have their own biases.*

*We would not recommend combining cold and warm dissociations as without profiling the warm and cold fractions separately it would be near impossible to deconvolute biases.*

*We recommend that users test and weigh up these options before committing to a large single cell profiling project. Informed choice of one protocol will minimise biases.*

Q 2-2. Current work supports the general findings of the single cell and Human Cell Atlas community and broadly reiterates that different tissue preservation, dissociation, and preparation methods tested each have pros and cons, making them all potentially usable under different scenarios.

*A 2-2. We agree with the reviewer. We would like to stress that while broadly speaking all methods indeed have their pros and cons, it is crucial to have an idea of what these pros and cons are (and the relative level of bias each introduces) and how they compare to available alternatives. Our study is an important resource of such knowledge where several alternative protocols are compared side by side.*

Q 2-3. When this is combined with the fact that this data may be hard to extrapolate to different tissues it can be difficult to make definitive conclusions about the representative fraction of each cell type present in the mouse kidney through transcriptomic data alone.

Given that one can expect heterogeneity between individual mice, sex-specific differences, etc. it is difficult to conclude anything from the current study other than that are many confounding factors.

*A 2-3. The reviewer points out a valid concern. The experiments in this study were designed in a way minimising confounding factors between samples that were compared in each section of the manuscript. For instance, for tissue dissociation protocol comparison, only male mice were used, all from the same litter. In addition, three kidneys were used as biological replicates to mitigate the differences between individual mice. Another example, comparison of fresh and preserved single-cell suspensions was done for aliquots of the exact same suspensions, hence, there is no heterogeneity between individual mice or sex-specific differences that could confound the results.*

Q 2-4. It is unclear as to how to interpret the results in context of mouse kidney (e.g., for all the different cell types % reported here, which one should we go with?) or translate the finding to human kidney, mouse strains, pathologies, etc.

*A 2-4. We focused on mouse kidney as it is simple to reproducibly sample the same organ and that there was a wealth of complementary data to compare our data to. Regarding kidney cell type composition we stated the following in the Results section:*

*“We next compared the observed cell composition to estimates of epithelial cell type contribution based on quantitative renal anatomy, as reported by Clark et al. recently [32]. Fig. 4B and Supplementary Fig. 9 show that for some cell types, such as podocytes, scRNA-seq yields proportions most similar to the quantitative renal anatomy estimates, whereas for other cell types, such as loop of Henle cells, snRNA-seq better captures cell composition.”*

Q 2-5. How well do the current findings in tissue dissociation translate to other tissue types? How do they translate to diseased tissue? Can the results be generalizable to human tissue?

For example, there is growing evidence that there is loss of epithelial cells under cryopreservation, loss of immune cells in single nuclei protocols, are likely to hold true in other tissue/organs. Others (PT vs. aLOH cells) are likely not applicable outside the context of mouse kidney.

*A 2-5. The reviewer raises a very important issue, which we certainly did not discuss enough in the manuscript.*

*We started this work after spending substantial amounts of time and money on 10x experiments profiling diverse tumour samples with rather disappointing returns on investment. For some tumour types we observed ‘good’ data with somewhat expected cellular populations, for others, either the cellular proportions did not match the expectations of our collaborators, the cell viability was poor, or the numbers of genes detected low. This resulted in us taking a step back and designing a controlled experiment, systematically comparing what was reported as ‘best’ in multiple papers.*

*From our experiments we find/confirm:*

*1.	Depletion of epithelial cell types (PT cells) and other kidney-specific epithelial cells in cryopreserved dissociated cell samples. As the majority of human cancers are epithelial in origin this has direct relevance to human cancer biology.*

*2.	Under-representation of T, B and NK cells in snRNA-seq data. Again this is of critical importance for studies of tumour immunology and studies looking at immunotherapies.*

*3.	Given these biases we as a community need to make informed choices for specific experiments and keep working to develop methods with fewer biases.*

*Within our collaborative network we are using these results to guide our collaborators. We are instructing our collaborators to use cold dissociation and freshly profile samples where possible. For clinical samples (where we often need to wait for pathology results) we discuss with the collaborators the different biases of cryopreservation, methanol fixation, or snRNA-seq from frozen tissue. Where information on lymphocytes (T, B, NK) is required we do not recommend snRNA-seq.*

*To reflect the relevance to human disease biology we have replaced the conclusion with the following:*

*“From our experiments we have confirmed several observations by others in the field that have direct relevance to designing single cell experiments studying human disease biology. Specifically, the depletion of epithelial cell types in cryopreserved samples is concerning given the majority of human cancers are epithelial in origin. Similarly, the under-representation of T, B and NK cells in snRNA-seq data is of critical relevance for studies of tumour immunology, tumour immunotherapy and autoimmune disease. By comparing these protocols across a single system (mouse kidney) we are now using these results to guide our collaborative network on the best approach for their circumstances. We are instructing our collaborators to use cold dissociation and freshly profile samples wherever possible. For clinical samples (where we often need to wait for pathology results, or are constrained by how archival material has been stored) we discuss with the collaborators the different biases of cryopreservation, methanol fixation, or snRNA-seq from frozen tissue and together decide the best approach. Where information on lymphocytes (T, B, NK) is required we do not currently recommend snRNA-seq. Researchers will undoubtedly continue to develop new methods aimed to reduce bias. Given the high cost of these experiments it is critical that these methods are systematically compared to allow the community to decide which to adopt.”*

Q 2-6. I am unsure if the stress response score suggested by the authors is a good metric in that as it looks like it just takes the highest stressed cells in a given sample as 1, which doesn't mean that those cells are necessarily much stressed. As in, the high stressed cells might just be lowly stressed cells being compared to basically unstressed cells. It may be used to compare cell types present in a given sample but cannot be used to compare stress responses in a given cell-type across samples prepared under a set of tissue dissociation/preservation conditions, which would be more meaningful. How does the stress score proposed here compare to other scoring?

*A 2-6. The reviewer raises a valid point. The purpose of the stress-scoring procedure was to be able to quantitatively compare the behaviour of the cell types within this experiment. This is reflected in the following part of the Results section: “Notably, the numbers of differentially expressed genes varied among cell types (Fig. 2B), suggesting that cell types respond differently to warm tissue dissociation. To quantify these differences, we selected a set of 17 known stress-response-related genes that were induced in the warm-dissociated samples”. Hence, the gene list was data-driven and indeed, as the reviewer pointed out, measures how other cell types differ from the more stressed ones within this experiment. We did not use this gene list to decide on how stressed the cells with the high scores are. Similarly, this gene list was not intended to be used as is for other tissues. Hence, we believe, the scoring procedure is appropriate for its purpose.*

*We explicitly list the corresponding genes in several places in the manuscript, so that readers who are interested in comparing these genes to those seen in their tissue could easily access them. According to Tabula Muris findings, “organs likely display unique dissociation-related expression profiles”, hence, we think claiming our gene list to be a universal one and encouraging researchers to use it as is for their data would be unjustified.*

*As to whether stressed cells are stressed enough to be considered being stressed. We performed differential gene expression analysis for bulk RNA-seq profiles of single-cell suspensions. Supplementary Table 1 lists genes with thresholds of logFC > 2, FDR < 0.05 (which cannot be called particularly permissive, yet identifies many known immediate-early genes) as well as results from gene set enrichment analysis, where top significant GO BPs are “regulation of cell death”, “regulation of apoptotic process”, “regulation of programmed cell death”. This appears to us as a detectable stress response. Supplementary Table 3 shows similar data for scRNA-seq.*

Q 2-7. In discussion, there is no clear conclusion from the different conditions tested- the reader would expect some guidance from the authors in interpreting the multiple datasets generated under different conditions. Each seems to have pros and cons depending on which cell type(s) one might be interested in. Some discussion about the uses and pitfalls of both might be appropriate.

*A 2-7. We have rewritten the conclusion (see response to point 2-5) to give recommendations to the pros and cons.*

Q 2-8. So there are differences: how to correct for them? There are different computational methods (Canonical Correlation Analysis, Mutual Nearest Neighbor, k-nearest-neighbor batch-effect test, etc.) to correct for batch effects and other technical artifacts that are not tested or compared here. Can they be used to combine the different datasets generated from the different techniques tested) be to generate a consensus data-set? This may fix the shortcomings of the different preservation/dissociation/preparation methods? Also, for the cells that are preferentially adversely affected by the preps, can the stress response genes or modules be regressed out?

Computational techniques to address such issues are not used on this well-designed experiment. Other than the use of scMatch, computational analyses were insufficient. Much more can be learned from such a carefully planned, exhaustive set of experiments. Why not?

*A 2-8. We agree with the reviewer that this dataset is well-suited for benchmarking of various computational tools that combat the reported artefacts and we have made the data open to the research community for exactly that reason.*

*Beyond making the manuscript substantially larger, there are several reasons as to why such benchmarking of computational methods was not a part of the present manuscript:*

*1.	Well-conducted benchmarking of computational methods for such corrections typically form a large and independent study on its own. For instance, one recent study focusing only on batch-effect correction methods (and not other technical artefacts) had to evaluate 14 different methods (PMID: 31948481, published in Genome Biology earlier this year).*

*2.	The main motivation behind this study was to reveal biases of some of the existing wet-lab protocols. In our view, this would guide and help researchers in two main aspects: 1) choice of the protocol before the experiment and 2) understanding of possible artefacts in the data from already completed studies. As analysts we all know avoiding introduction of biases into a dataset is preferable over computational methods to mitigate them.*

*3.	Importantly, trying various computational methods and reporting on their performance would not change the conclusions of the present manuscript regarding the artefacts of the wet-lab protocols.*

*4.	We make our data publicly available as raw fastq files and as filtered count table, and we believe that the use of this data in the future comprehensive benchmarking studies of computational tools will be much more conclusive and valuable for the community rather than selective testing of only a handful of methods that we would be able to accomodate into this already lengthy manuscript.*

*Nevertheless, we agree that the reviewer has raised an important point and we added the following to the discussion:*

*“In this study we reported on a range of biases of experimental procedures. We would like to stress that computational tools are being actively developed to mitigate these biases. These include tools for decontamination such as DecontX [PMID: 32138770] or SoupX [doi.org/10.1101/303727], tools for batch-effect removal such as Seurat [PMID: 29608179] and Harmony [PMID: 31740819] and many others. Hence, even when a dataset in hand is affected by pronounced technical artefacts, certain computational techniques might be useful in reducing their effect.”*

Q 2-9. A lot of the cell numbers we see comparing techniques don't come with comments on significance or values to show significance. In the absence of conclusive numbers for cell-type representation or expression, alternative techniques like FACS sorting on dissociated cells, or IHC on tissues may be used as orthogonal approaches to establish cell-type % and their gene expression.

*A 2-9. The significance testing of differences in cell-type representation and expression are included throughout the manuscript. Differences in cell-type representation are tested using two-sided chi-square test. Differences in expression are tested using Wilcoxon test.*

*The reviewer raises an important point about orthogonal approaches also raised by reviewer 1 (Q 1-9). We repeat the response to reviewer 1 here.*

*We added the following sentence to the Discussion section:*

*“It is important to remember that cell type composition of single-cell or single-nucleus data differs from that of the original tissue and complementary approaches may be considered to improve the accuracy of the estimates. We note that even ISH/IHC/spatial transcriptomic methods, unless carried out on sufficient serial sections to completely survey the tissue of interest, will only give cellular proportions for the sections examined and will not give a whole (3D) organ estimate. Similarly, flow cytometry would help as an orthogonal approach to quantify cellular proportions within single cell suspensions but still requires the organ to be dissociated, which as we have shown here has biases. Although in vitro cell mixtures, or cell spiking experiments could be used to introduce different cell types at known ratios, we have not employed them in these experiments as they do not represent a true scenario of dissociation from a solid tissue”*

Q 2-10. Of the stress response genes identified in the study, are there any that are specific to the kidney or are they generally applicable to other tissue types?

*A 2-10. This is an interesting point. First, we would like to note that we did not identify new stress response genes in this study. We started from a list of genes over-expressed in warm-dissociated cells and searched the literature for whether these genes are known to be stress-response-related. Naturally, literature-based evidence came from various tissues and conditions, hence, there is no reason to assume the genes to be kidney-specific. Of note, all of the 17 genes were also reported in the seminal work by van den Brink et al. in muscle (PMID: 28960196).*

Q 2-11. It is unclear if the cold dissociation is indeed less stressful or if the lower temperature is suppressing the transcriptional machinery in general. In parallel, it is unclear if the 37oC incubation per se in the warm dissociation caused stress response or it is the presence of the tissue/cells outside their native/ physiological environment that lead to stress response. 37C is after all normal physiological temperature.

*A 2-11. We thank the reviewer for the comment.*

*In our manuscript we mentioned that for cold dissociation “low temperature should minimise new transcription” whereas for warm dissociation we wrote that “At this temperature, the cell transcriptional machinery is active, hence, gene expression can be altered in response to the dissociation and other environmental stresses”.*

*We agree that the act of dissociation and removal from physiological conditions is the cause of the stress and that the 37˚C temperature per se is not causing the stress. What we can conclude however is that cold dissociation can mitigate changes in gene expression as the machinery is effectively stopped at low temperature.*

Q 2-12. Minor concerns: Unclear as to what the authors mean when they propose that the aLOH cells are being lost in the microfluidics (p5, l16). Please clarify.

*A 2-12. Our findings indicate that the proportion of aLOH cells in cell suspension before loading onto 10x Chromium (as measured by bulk RNA-seq) was higher than the proportion observed in the single-cell data. From this we conclude that these cells were undersampled from the cell suspension. We hypothesized that they might be lost on the microfluidics device (e.g. cells might be too large or have irregular shape which prevents them from being encapsulated efficiently). Another hypothesis is that they may be resistant to lysis in the 10x buffers, however we have no simple way to test this without first purifying aLOH cells.*

Q 2-13. How intact were the cells after running the tissue through the octoMacs? The octoMacs destroys tissue, so how are they ensuring that the cells are intact? Perhaps the octoMacs is shredding up more delicate cell types that they didn't catch?

*A 2-13. The Miltenyi gentleMACS octo dissociator is a common tissue dissociation equipment used in many labs around the world and has an optimised ‘mouse kidney’ program. There is indeed the possibility that the octoMacs damages delicate cell types; however, OctoMACS was used in both warm and cold tissue dissociation protocols and, thus, cannot be the reason for selective loss of certain cell types.*

Q 2-14. What was the viability of the cells?

*A 2-14. Thank you for the suggestion. We have now added this information to Additional file 18.*

Q 2-15. - How did the authors identify the stopping point for the different enzymatic digestions? Are there any guidelines for visually distinguishing cells from debris during dissociation?

- How did the authors identify the end point of tissue digestion? Were different incubation times tested?

*A 2-15. For warm dissociation, we followed the commercial protocol and did not adjust the time. We didn’t spot any issues (e.g. undigested bits of tissue) with the protocol so did not see a need to change the commercial protocol.*

*For cold dissociation, we followed the methods in the Adam et al publication but extended the digestion time as we could still see undigested tissue. The end point of digestion was confirmed by no visible tissue chunks and checking single cell suspensions under the microscope.*

*We thank the reviewer for raising these important questions and we now have added the following sentence to the Methods sections on warm and cold dissociation protocols:*

*“Complete tissue dissociation was checked by examining under the microscope and confirming the absence of visible tissue chunks.”*

Q 2-16. Did they filter out any undissociated clumps during their cell/nuclei prep steps?

*A 2-16. Yes this is described in the methods. Cells went through a 70um filter and then a 40um one. Nuclei preps went through either a 40um or 30um filter depending on whether protocol 1, 2 or 3 was used. For nuclei preparations, there was very little on the surface of the filter once it had gone through. For cell preparations, there was some material on the surface of the filter, but we have not observed anything concerning such as chunks of undissociated tissue.*

Q 2-17. Why not any other FBS/DMSO mixes? Is the epithelial cell loss consistent across all freezing media?

*A 2-17. We agree that an alternative cryostorage media may solve this and before COVID-19 caused the shut down of our lab we were starting experiments to test 90% FBS (often recommended for sensitive cell types) and specialised commercial cryostorage media. Unfortunately we have been told to expect a shutdown of months not weeks so we are unable to incorporate these into the current manuscript. In absence of these additional experiments, we comment that we chose 10% DMSO and 50% FBS as it is used broadly by the community and is very similar to the 40% FBS recommended by 10x (10x Genomics, CG00039, Rev D) (as per our Discussion section). The reader is also informed of two other studies in the discussion where both 90% FBS and 10% FBS yielded ‘good’ recovery of cells.*

Q 2-18. Optical microscopy images of the final cell/nucleus suspensions obtained from each of the techniques tested would be very useful to visually assess the diversity in cell types, presence of debris, etc.

*A 2-18. We agree with the reviewer. In hindsight we should have collected these data systematically, unfortunately, we don't have comprehensive high quality images at the same resolution and at all steps (before and after filtration, storage etc.). For some we have only DAPI stained images captured on the countess while others we have DAPI and brightfield. These images are ok for cell/nuclei quantitation but are low quality. Additionally we are currently transitioning from using a countess to a cellometer with different imaging, as we have found the countess cell count estimates to be unreliable. If the reviewer would like to see this we can send an additional supplementary file but we don’t think it is high enough quality to draw any conclusions.*

Q 2-19. Reasoning behind "higher fraction of recovered PT cells (7.65% vs. 0.57%) in the repeated experiment" (pg 12, l2) is hand-wavy. Perhaps the effect of worse chemistry in v2 (that would translate to lower capture before capture) can be simulated with down-sampled data and compared?

*A 2-19. Although it is feasible for us to randomly down-sample the v3 data to the same number of UMIs as in the v2 data, this assumes that there is a linear relationship between measurements using v2 and v3 chemistry. This does not appear to be the case: https://kb.10xgenomics.com/hc/en-us/articles/360026501692-Do-we-see-a-difference-in-expression-profile-of-3-Single-Cell-v3-chemistry-as-compared-to-v2-chemistry”*

Q 2-20. Ambient RNA contamination can be checked with 'EmptyDrops' analysis (DropletUtils package).

*A 2-20. In this study we indeed used EmptyDrops to distinguish real cells from ambient RNA contamination. This is mentioned in the “scRNA-seq and snRNA-seq data processing” section of the Methods*

**Second round of review**

**Reviewer 2**

Thank you for careful consideration and clarifications made to the manuscript based on reviewer comments
